# Multiple faces of stress in the zebrafish (*Danio rerio*) brain

**DOI:** 10.3389/fphys.2024.1373234

**Published:** 2024-04-15

**Authors:** Constanze Pietsch, Jonathan Konrad, Elena Wernicke von Siebenthal, Paulina Pawlak

**Affiliations:** ^1^ School of Agricultural, Forest and Food Sciences (HAFL), University of Applied Sciences Bern (BFH), Zollikofen, Switzerland; ^2^ Division of Behavioural Ecology, Institute of Ecology and Evolution, University of Bern, Bern, Switzerland

**Keywords:** aquaculture, stressors, gene expression patterns, machine learning, classification

## Abstract

The changing expressions of certain genes as a consequence of exposure to stressors has not been studied in detail in the fish brain. Therefore, a stress trial with zebrafish was conducted, aiming at identifying relevant gene regulation pathways in different regions of the brain. As acute stressors within this trial, feed rewarding, feed restriction, and air exposure have been used. The gene expression data from the experimental fish brains have been analyzed by means of principal component analyses (PCAs), whereby the individual genes have been compiled according to the regulation pathways in the brain. The results did not indicate a mutual response across the treatment and gender groups. To evaluate whether a similar sample structure belonging to a large sample size would have allowed the classification of the gene expression patterns according to the treatments, the data have been bootstrapped and used for building random forest models. These revealed a high accuracy of the classifications, but different genes in the female and male zebrafish were found to have contributed to the classification algorithms the most. These analyses showed that less than eight genes are, in most cases, sufficient for an accurate classification. Moreover, mainly genes belonging to the stress axis, to the isotocin regulation pathways, or to the serotonergic pathways had the strongest influence on the outcome of the classification models.

## 1 Introduction

Distress is defined as a condition that interferes with the well-being of an animal (Ihwan et al., 2016; Balasch and Tort, 2019). Fish in aquaculture and research facilities are often subjected to distress not only for short time periods (acute stress) but also for longer ones (chronic stress). However, rearing fish under conditions that respect the species-specific needs of the animals improves their well-being and performance in fish farms (Macaulay et al., 2022) and under experimental conditions (Grunow and Strauch, 2023). The main question of the present work is to identify which set of genes in which brain region is characteristic of specific stress situations and should therefore be focused on in future fish welfare assessments. Accordingly, brain gene expression studies have increasingly investigated the responses of the brain parts separately (Vindas et al., 2018; Huang et al., 2019; Martorell-Ribera et al., 2020). The functions of the telencephalon have long been regarded solely to be olfactory (Aronson, 1967), but more recent research has confirmed its important role in the expression of emotional and motivational behavior and in stress responses in teleosts (Lau et al., 2011; von Trotha et al., 2014; Pawlak et al., 2022). In addition, the hypothalamus plays an important role in energy homeostasis and appetite regulation, which may also be affected by stress (Pawlak et al., 2023). The optic tectum not only is essential for visually mediated behaviors in teleosts (Kramer et al., 2019) but also receives input from other brain areas, such as the hypothalamus and cerebellum (Heap et al., 2013; Filosa et al., 2016). The cerebellum of fish is responsible for the coordination of body movements (Moens and Prince, 2002) and has been linked to spatial navigation (Duràn et al., 2014), and also appears to play a role in social stress in fish (Guo et al., 2022).

The expression analysis of genes involved in stress responses in fish commonly includes genes of the hypothalamus–pituitary–interrenal (HPI) axis. The excitation of pro-opiomelanocortin (POMC)–expressing neurons in the hypothalamus results in the activation of corticotropin-releasing factor (CRF) neurons and the release of CRF (Cerdá-Reverter et al., 2003; Sanchez et al., 2009). The role of CRF in zebrafish exposed to stress, e.g., by confinement, has been investigated by Ghisleni et al. (2011). Urotensin 1 (URO1), which is structurally related to the tetrapod urocortin, is a paralogue of the CRF and has the ability to bind to the CRF receptors r1 and r2 (Suda et al., 2004). The binding protein for CRF (CRH-BP) rather co-occurs with CRH than with URO1 in the adult zebrafish brain, reducing its availability and thus limiting further release of the adrenocorticotropic hormone precursor POMC (McClennen et al., 1998; Alderman and Bernier, 2007). The downstream production of cortisol allows the activation of glucocorticoid receptors (GRs) and mineralocorticoid receptors (MRs), which further regulate other pathways in some cases in a ligand-specific way (Pippal et al., 2011; Cruz et al., 2013; Lee et al., 2019). However, cortisol also influences vasotocin and isotocin secretion in gilthead sea bream, *Sparus aurata*, and round goby, *Neogobius melanostomus* (Kalamarz-Kubiak, 2018). In the case of isotocin, it has been assumed that a non-genomic pathway mediates the effects of cortisol (Kalamarz-Kubiak, 2018). Furthermore, isotocin signaling in fish is involved in social behaviors, stress responses, and aggression (Semsar et al., 2001; Salek et al., 2002; Kulczykowska et al., 2009; Pawlak et al., 2023).

Besides measuring the activity of HPI axis–related genes, brain activity can also be assessed, for example, by using the activation of immediate early genes (IEGs) indicating neuronal activity. Several IEGs are known, with C-FOS being one of the most frequently analyzed IEGs. So far, light avoidance (Lau et al., 2011), sleep and waking behavior of zebrafish (Chatterjee et al., 2015), activation of the reward system (von Trotha et al., 2014), and caffeine have been shown to involve changes in *c-fos* expression in the zebrafish brain (Ashlin et al., 2018). Furthermore, IEGs also include factors for neuronal development (EGR-1 and ERK; Randlett et al., 2015) or neurogenesis (NeuroD; Alfonso et al., 2019) and for controlling the cytoskeleton, protein synthesis (e.g., CKAP5 or the eukaryotic initiation factor 2, EIFA), or metabolic enzymes, such as glyceraldehyde-3-phosphate dehydrogenase (GAPDH), pyruvate kinase (PYRKIN), citrate synthase (CITRSYN), or succinate dehydrogenase (SUCCDH; Pawlak et al., 2022). In addition, changes in the energy status can also lead to the regulation of the mechanistic target of rapamycin (mTOR). In cavefish brains, *mTOR* expression has been shown to increase with the application of the appetite-stimulating factor ghrelin (GHR) but not with injections of the anorexigenic factor cholecystokinin (CCK; Penney and Volkhoff, 2014).

Stress influences appetite regulation and feeding behavior in teleosts (Höglund et al., 2007; Leal et al., 2011; Pawlak et al., 2023); therefore, appetite-regulating genes have also been included in the present study. Furthermore, it has been shown that the administration of dopamine decreases gene expression of the agouti-related protein (*agouti*) but not of *pomc* and neuropeptide y (*npy*; He et al., 2018). The agouti-related protein family is known to centrally regulate food consumption in zebrafish (Shainer et al., 2019). In addition, two paralogs of the agouti signaling proteins have been identified in the zebrafish brain, which differ in their tissue distribution and function (Shainer et al., 2019).

The serotonergic system is widely distributed in the fish brain, and the functions of serotonin (5-HT) in the zebrafish brain have been described by Lillesaar (2011). Serotonin uptake variations influence anxiety and modulate the vocal–acoustic circuitry and behavior in teleosts (Simmons et al., 2017; Timothy and Forlano, 2020) and may also play a role in stress responses (Pawlak et al., 2023). Furthermore, the serotonin precursor tryptophan affects serotonin signaling and interferes with stress-induced anorexia in the rainbow trout, *Oncorhynchus mykiss* (Höglund et al., 2007; 2019). Interestingly, it has also been reported that darkness activates the majority of the tryptophan hydroxylase 2 (TPH2) serotonergic neurons within the raphe of zebrafish, thus further supporting the possibility that the release of serotonin is involved in aversive behaviors (Cheng et al., 2016). Being the rate-limiting step in serotonin formation, the activity of TPH in fish has been assumed to be a specific marker of 5 HT-producing neurons (Chivite et al., 2021). Interestingly, *in vitro* treatment of the telencephalon of tilapia (*Oreochromis mossambicus*) with noradrenaline and serotonin was found to stimulate the release of CRF (Pepels et al., 2004). Similarly, it has been shown that tyrosine hydroxylase (TH) is considered a marker for dopamine activity in tilapia and is related to the repeated exposure to aquaculture-relevant stressors such as handling, chasing, netting, and lowering of the water levels (Chabbi and Ganesh, 2015). In addition, the opioid system is known to be involved not only in nociception and analgesia in higher vertebrates but also in stress responses and reward processing (Le Merrer et al., 2009). Increasing evidence has shown that stress-induced analgesia also exists in zebrafish (Thomson et al., 2020). Furthermore, acute stress affecting appetite regulation is in parallel to the changes in the expression of opioid receptor delta in common carp (Pawlak et al., 2023).

Thus, the present study concentrates on the primary stress responses at the brain level to clarify which set of genes expressed in different brain regions is characteristic of specific stress situations. The investigations further reflect on whole-body steroid levels as the subsequent level of stress responses.

## 2 Materials and methods

### 2.1 Rearing conditions

**FIGURE 1:**
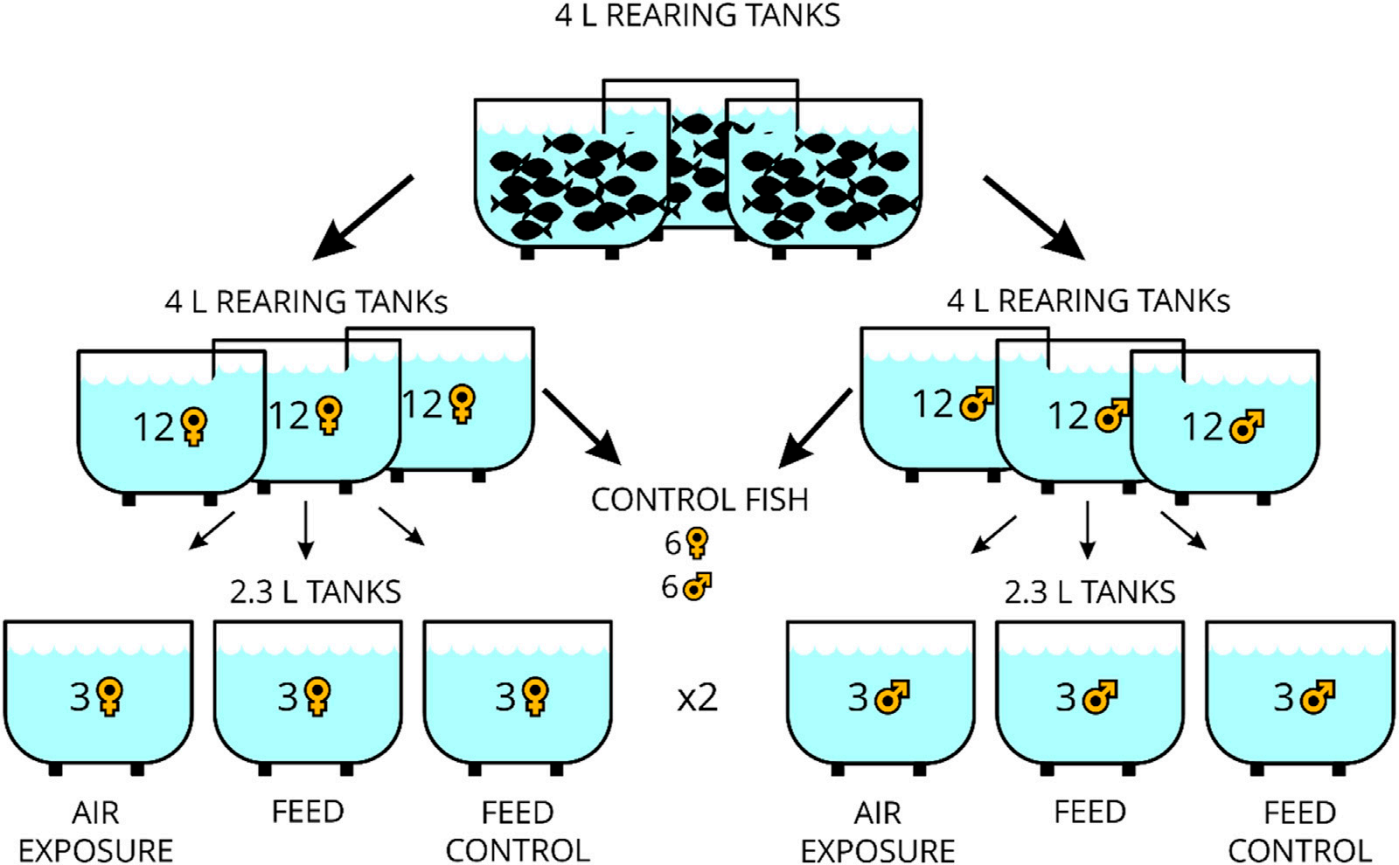
Overview of the experimental design of the study.

Four-liter rearing tanks (MC2B, dimensions: 332 × 150 × 130 mm, Zebcare by Fleuren & Nooijen BV, Nederweert, NL) for fish breeding were part of a recirculating aquarium system equipped with a biofilter. In these, the zebrafish (*Danio rerio*) of the wild-type × AB strain (ZIRC, Eugene, OR, United States) were 6–12 months old and fed twice daily with freshly hatched *Artemia* nauplii (Ocean Nutrition Europe, Essen, Belgium) in the morning between 8:00 and 8:30 a.m. and in the afternoon with commercial dry feeds (Zebrafeed, 0.2–0.4 mm size, from Sparos, Olhão, PT). From these fish, 24 individuals (male and female fish, separately) were transferred to individual rearing tanks with an effective volume of 2.7 L (MC.1 tanks, dimensions: 235 × 150 × 130 mm, Zebcare by Fleuren & Nooijen BV, Nederweert, NL) for 3 days in a smaller recirculating aquaria system (DC-96 benchtop system, Zebcare by Fleuren & Nooijen BV, Nederweert, NL) with continuous monitoring of the water temperature, pH, and conductivity levels (JUMO AQUIS touch S, JUMO AUTOMATION GmbH, Eupen, Belgium) and used for the stress experiment thereafter. Water temperature, dissolved oxygen, salinity, and pH in the experimental tanks were manually checked at each sampling day using a mobile device (HQ40D, Hach-Lange GmbH, Rheineck, Switzerland), which showed stable values averaging at 26.1°C, 7.40 mg/L, 571 μS, and 7.38, respectively. Ammonium, nitrite, and nitrate content in water were measured on each of the three sampling days, and mean values of 0.01 mg/L, 0.01 mg/L, and 13.5 mg/L have been noted. The light cycle in the facility was 14 h light:10 h dark. The experimental design of the study is presented in Figure 1. The six control fish reared for 3 days as mentioned above were taken directly from the rearing tank before the feeding time in the morning of the same day as the treated fish. The remaining fish were placed in individual tanks in groups of three fish in duplicate, reared for 3 days without disturbance, and remained unfed in the morning. For the “air exposure” treatment, the fish were exposed in a net to air for 1 min and euthanized 60 min after that. For the “feed” treatment, three fish were similarly housed together for 3 days in duplicate and fed the expected *Artemia* nauplii ration in the morning, whereas for the two “feed control” treatments, three fish were not fed at the expected time in the morning. Euthanasia was performed with an overdose of tricaine methanesulfonate (>150 mg/L MS-222, Sigma-Aldrich, Buchs, Switzerland) for less than 2 min. The weight of the fish (Mettler Toledo GmbH, Greifensee, Switzerland) and the total length and standard body length were noted, and Fulton`s condition factors were calculated by dividing the weight of the fish by the total length cubed × 100 (Fulton, 1902). In addition, the body mass index was calculated [weight (g)/standard length (mm)^2^] for each individual. The brains were excised and stored in RNAlater^®^ (Sigma-Aldrich, Buchs, Switzerland) for at least 24 h and then cut into four parts (tel = telencephalon, hyp = hypothalamus, opt = optic tectum, and rhomb = rhombencephalon comprising the corpus cerebelli and medulla oblongata) using a microscope (VWR^®^ VisiScope^®^ STB150, Stereo Microscope, VWR International, Switzerland). All experiments/procedures were approved by the local animal care committee (license no. BE69/2020) and were in accordance with guidelines set by the Swiss Council on Animal Care.

### 2.2 PCR conditions

Primer pairs used for the gene expression studies have been prepared with Primer3 (Untergasser et al., 2012), and the individual sequences are given in Supplementary Table S1. Prior to the analyses, all primers were validated, and the respective PCR products were confirmed by Sanger sequencing performed by Microsynth AG (Balgach, Switzerland) after the cleanup of the PCR products with suitable kits (NucleoSpin Gel and PCR Clean-up, Macherey-Nagel AG, Urdorf, Switzerland). The sequencing results have been compared to known sequences via the basic local alignment search tool (Blast-N) function from the National Center for Biotechnology Information (NCBI, https://www.ncbi.nlm.nih.gov/). The primer efficiencies were analyzed using LinRegPCR (version 2020.2; available at https://medischebiologie.nl/files/) and are reported in Supplementary Table S1. All amplicons had a size ranging from 110 bp to 200 bp. The total RNA from each of the four parts of each brain (tel, hyp, opt, and rhomb) was extracted using the RNeasy Micro Kits (QIAGEN AG, Hombrechtikon, Switzerland) with an on-column DNase I treatment. The RNA content was confirmed using a microplate for a plate reader (Tecan Infinite M200 Pro, Tecan Instruments, Crailsheim, Germany). Subsequently, total RNA was reverse transcribed into cDNA using the ProtoScript Reverse Transcription Kit (NEB, distributed by BioConcept AG, Allschwil, Switzerland) according to the manufacturer’s instructions. Thereafter, the cDNA content was adjusted to 75 ng per ul using nuclease-free water (Ambion^®^, distributed by Thermo Fisher Scientific, Switzerland) and used for pre-amplification with 18 cycles using the PreAmp Master Mix from Fluidigm Corporation (South San Francisco CA, United States) and a pre-mixed primer solution, followed by an exonuclease treatment (BioConcept AG, Allschwil, Switzerland) according to the manufacturer`s protocol. Subsequently, the pre-amplified cDNA was diluted fourfold, mixed with the SsoFast™ EvaGreen® Supermix with Low ROX master mix (Bio-Rad Laboratories AG, Cressier, Switzerland), and the Fluidigm sample reagent (Fluidigm Corporation, South San Francisco, CA, United States) according to the manufacturer’s protocol, loaded on BioMark™ IPC chips (192 × 24, Fluidigm Corporation, South San Francisco, CA, United States), and incubated in a Juno system for 33 min. Thereafter, real-time PCRs were run using a BioMark™ system (Fluidigm Corporation, South San Francisco, CA, United States). The initial results were calculated using the Fluidigm Real-Time PCR Analysis software (version 4.8.1, Fluidigm Corporation). The optimal reference genes were extracted from a set of 10–11 potential reference genes by using the RefFinder (http://blooge.cn/RefFinder/) separately for each brain part. The results from the reference gene evaluation can be found in Supplementary Table S2.

All gene expression values were calculated relative to the expression of the selected reference gene (dCt method) and were further calculated as fold changes compared with the respective controls. Only significant gene expression differences are shown in Figures 3, 4.

### 2.3 Analysis of body corticosteroid levels

Prior to the steroid analyses, diethyl ether extractions of whole-body homogenates (without the heads) were repeated twice; the extracts were purified on C18 columns (Sep-Pak Vac C18 1 cc 100/BX, Waters AG, Dättwil, Switzerland), and the cortisol and cortisone levels were analyzed using a UHPLC–MS/MS system, as previously described (Pawlak et al., 2022). The final values were calculated as nanograms of steroid per gram of body weight.

### 2.4 Calculations and statistics

The body steroid levels (Pietsch, 2024) were log-transformed and used for two-sided ANOVA tests with subsequent Tukey HSD tests to compare the groups with each other. For the correlation analyses, the log-transformed cortisone and cortisol levels were compared with Pearson correlation tests in RStudio (version 1.2.1335). The gene expression data investigated in the different brain parts (Pietsch, 2024) were used to build mixed models with a fully Bayesian approach using the brms package (Bürkner, 2017) in RStudio. Based on pre-checks, t-distributions were chosen for the models with 10,000 iterations that included gene-specific random effects for the constants (α), gene-specific random effects for differences between groups (β), and animal-specific random effects for the constants (γ), as explained earlier by Burren and Pietsch (2021). Improved handling of possible outliers was achieved by posterior predictive checks based on Markov chain Monte Carlo (MCMC) approximation. The resulting replicated data under the fitted model were subsequently compared to the observed data. Finally, point estimators, their SEMs, credibility intervals, and posterior predictive *p*-values were calculated. Significance was determined using Wald χ^2^-statistics for generalized linear models and F-statistics for mixed models, and the estimated marginal means were calculated, which included their corresponding 95% credible intervals (95% CI). A *p* < 0.05 was considered statistically significant.

The genes investigated in the different parts of the brain were also subjected to a principal component analysis (PCA) in order to show gene clusters that typically respond to the distinct treatments. The PCA was run in RStudio on individual sets of genes (HPI axis–related genes, IEGs, appetite genes, and the remaining genes related to different pathways in the brain) to prevent a low sample-to-item ratio that would lead to incorrect factor structures and misclassification of items. Finally, the most contributing genes were compiled for each pathway, and a PCA run for these in female and male fish separately. Only the first two components of each PCA were used to prepare figures of the cos^2^ values for each brain region. For the female fish, the two-dimensional PCAs explained 76.6% of the variance in the telencephalon, 64.6% in the hypothalamus, 70.9% in the optic tectum, and 78.0% of the variance in the rhombencephalon. For the male fish, the two-dimensional PCAs explained 79.8% of the variance in the telencephalon, 75.9% in the hypothalamus, 73.1% in the optic tectum, and 78.1% of the variance in the rhombencephalon. More details on the contribution of each gene to the total variance for the individual treatment groups are given in the Results section.

The classification and feature importance calculation required bootstrapping of the data (Pietsch, 2024). For this, the gene expression values as log 2 of the normalized expression were used to generate bootstrapped data sets, with n = 100 data points separately for each treatment group and gender, for each gene using the R package boot (Canty and Ripley, 2021) and resampled (Hesterberg, 2015) in RStudio, and subsequently, random forest models were performed in a Jupyter Notebook based on Python 3. Accordingly, 70% of the data points from each bootstrap iteration was used for training the models, while the remaining 30% was used for model evaluation. The gene expression data were stratified and scaled prior to model building. The built-in feature importance function was used to report the contribution of the individual parameters to the model output. The number of genes that were sufficient to yield the same accuracy as the full data set for each brain part was calculated in an iterative process.

## 3 Results

### 3.1 Morphological characteristics of the fish and body steroid analyses

The initial analyses of the present study concentrated on the morphological and stress hormone differences between the experimental groups and differences between the female and male fish. The adult zebrafish that were used for the experiment showed significant differences in weight between the genders, which also affected the condition factor and BMI between female and male fish (Supplementary Table S3). An effect of the treatment group assignment or an interaction of treatment and gender was not observed.

**FIGURE 2:**
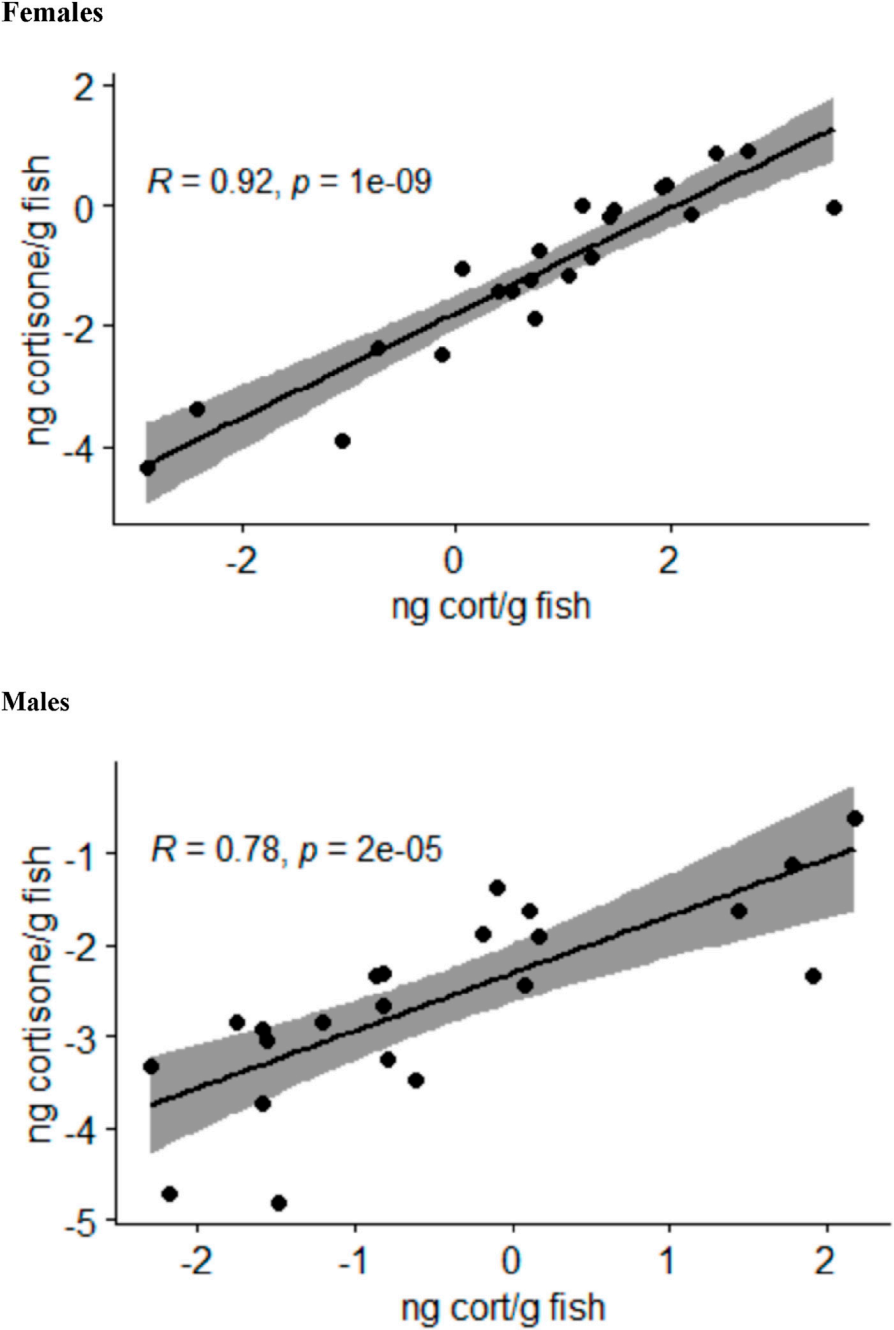
Relationship between the log-transformed whole-body cortisol (cort) and cortisone levels calculated as nanograms per gram of fish in female and male zebrafish across all treatment groups analyzed by Pearson correlation tests; the area shaded in gray indicates the 95% confidence interval.

The analysis of the body stress hormone levels revealed that the levels of 11-deoxycorticosterone and corticosterone in the whole-body samples were not quantifiable. The cortisol and cortisone levels for each fish correlated well with each other in female and male fish (Figure 2). Moreover, the two-sided ANOVA indicated a significant effect of gender (*p* = 0.005) and treatment (*p* = 0.012) on the steroid levels (Supplementary Table S4), but there was no significant difference between the steroid levels between the treatments after correcting the *p*-values for multiple comparisons. Thus, the whole-body steroid levels do not appear to be valuable proxies for the stress scenarios used in the present study.

### 3.2 Immediate early genes (*cfos*, *eiFa*, *egr-1*, *erk-1*, *neuroD*, and *palld*)

**FIGURE 3:**
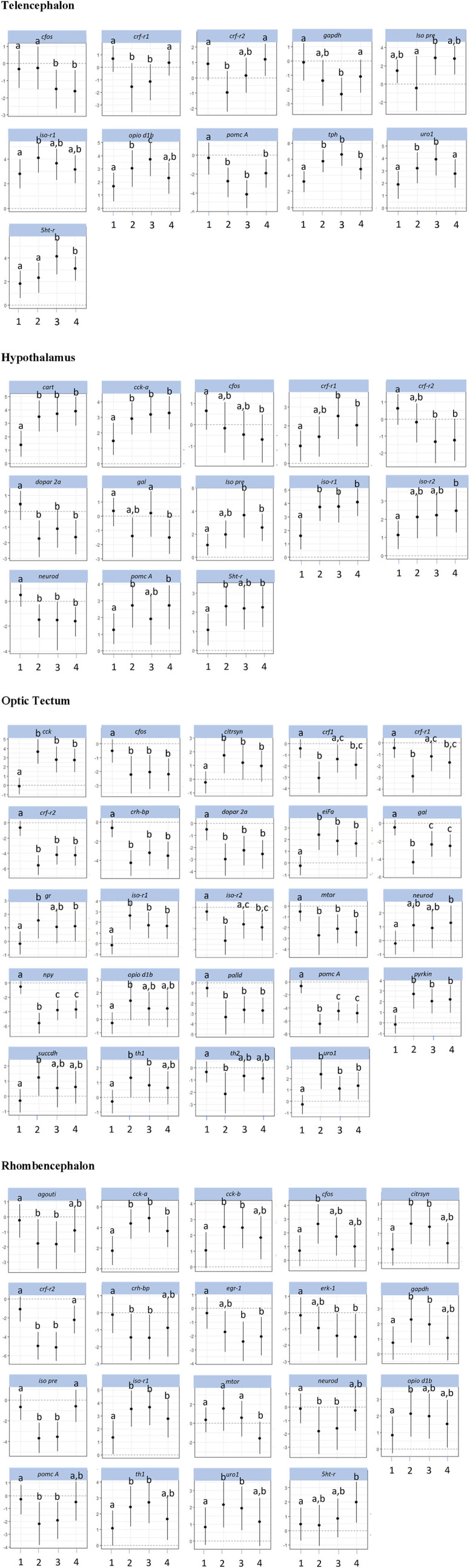
Gene expression profile in each of the four brain parts (telencephalon, hypothalamus, optic tectum, and rhombencephalon) in the female control fish (group 1) and fish at 60 min after the treatment (feeding = 2; feed contr = 3; and air exposure = 4); marginal means ± SEM; n = 6 per treatment; and the means of groups with the same letters are not significantly different from each other (*p* > 0.05).

**FIGURE 4:**
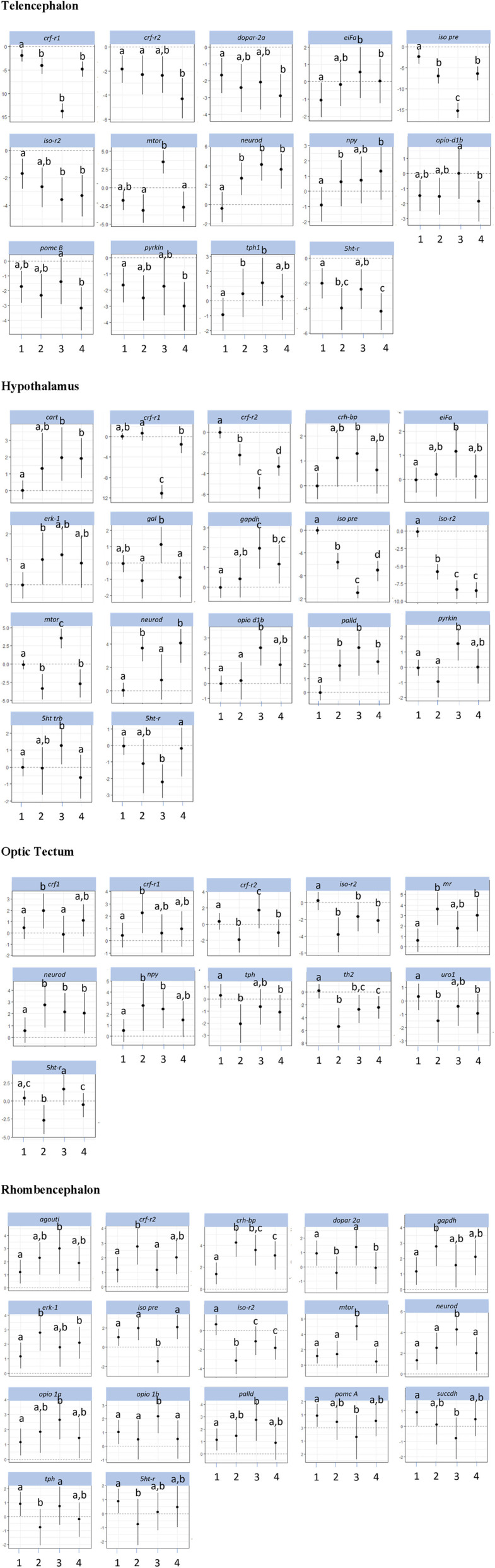
Gene expression profile in each of the four brain parts (telencephalon, hypothalamus, optic tectum, and rhombencephalon) in the male control fish (group 1) and fish at 60 min after the treatment (feeding = 2; feed contr = 3; and air exposure = 4); marginal means ± SEM; n = 6 per treatment; and the means of groups with the same letters are not significantly different from each other (*p* > 0.05).

The investigations of the gene expression patterns comprised four different brain parts in which different expression patterns in female and male fish were observed. In the telencephalon of female zebrafish, the expression of the IEG *c-fos* was lower in air-exposed animals and in the feed control fish compared with the controls (Figure 3, *p* = 0.012). A significant difference between control fish and feed control fish or air-exposed fish was also observed for the IEG *eiFa* in the telencephalon of male fish (Figure 4, *p* < 0.05).

For the hypothalamus of male fish, a higher mRNA expression of *eiFa* in fish of the feed control group was observed than the controls (Figure 4, *p* = 0.046). In addition, the expression of *erk-1* was higher in the feed-rewarded fish than in the control animals (*p* = 0.046). The expression of *palld* was higher in all male treatment groups than in control fish (*p* < 0.004). Moreover, the expression of *neuroD* in male fish was significantly higher in feed-rewarded and air-exposed fish than in the control animals (*p* < 0.001) or fish from the feed controls (*p* < 0.028). Similar to the situation in the telencephalon, *c-fos* expression showed a significant difference in value between the hypothalamus of control fish and of female fish.

For the optic tectum, the *neuroD* expression in male fish was found to be significantly higher in all treatment groups than in the controls (Figure 4, *p* ≤ 0.038). In the optic tectum of female fish, the expression levels of *c-fos* and *palld* were significantly lower and the mRNA level of *eiFa* was significantly higher in all treatment groups than in control fish (Figure 3, *p* ≤ 0.018). The expression of *neuroD* in female fish was only different between the air-exposed zebrafish and controls (*p* = 0.03).

Similar to the pattern in the hypothalamus, the expression of *erk-1* was higher in the feed-rewarded fish than in the control animals in the rhombencephalon of male fish (Figure 4, *p* = 0.002) but also higher in the air-exposed fish (*p* = 0.042). In addition, the expression of *palld* was higher in the feed control group than in the controls (*p* = 0.048). The expression of *neuroD* was also higher in this group than in all remaining groups (*p* ≤ 0.048). In female fish, the expression of *c-fos* was higher in the feed reward group than in the controls (*p* = 0.002). In addition, the mRNA levels of *egr-1* and *erk-1* were significantly lower in the feed control and air exposure groups than in the controls (*p* ≤ 0.038). Moreover, female fish showed reduced *neuroD* expression in the feed reward and feed control groups compared with control fish (*p* ≤ 0.024).

Overall, the effects of the different stressors on the IEGs indicate that besides *c-fos*, a number of other IEGs are regulated. In addition, the effects are distributed to all four brain parts investigated separately.

### 3.3 Effects on HPI axis–related genes

The investigations of the HPI axis–related gene expression patterns also comprised the four different brain parts in which different expression patterns in female and male fish were observed. The *crf*-related genes in the telencephalon in male fish showed significantly reduced *crf-r1* mRNA expression in all treatments compared with control fish (Figure 4, *p* < 0.01). In female zebrafish, the expression of this gene was only reduced in the feed group and in the feed control group (Figure 3, *p* < 0.024). Moreover, the transcript levels of *crf-r2* in male fish were only lower in air-exposed fish than in the controls (*p* < 0.001) and feed-rewarded individuals (*p* = 0.02). By contrast, female zebrafish showed only a lower expression of *crf-r2* in the feed-rewarded animals than in the controls or air-exposed fish (Figure 3, *p* ≤ 0.006). Only in the female telencephalon, *uro1* showed higher mRNA expression levels in the feed reward and feed control groups (Figure 3, *p* < 0.03). In addition, the expression of *pomc B* was found to be different between feed control animals and air-exposed male individuals (Figure 4, *p* = 0.044). However, female zebrafish showed significantly lower *pomc A* expression in all treatments than in control fish (Figure 3, *p* < 0.02).

In the hypothalamus of male fish, the expression of *crf-r1* was significantly different between all treatment groups (Figure 4, *p* < 0.028) and also significantly lower in the feed control group than in the controls (*p* < 0.001). Moreover, the expression of *crf-r2* in this brain part in male fish was different in all treatment groups, which included the controls (*p* < 0.002). In the hypothalamus of male fish, the *crh-bp* mRNA expression was found to be different between the controls and feed control fish (*p* = 0.038). In female fish, the *crf-r1* transcript levels in the hypothalamus showed significantly higher values than in the control animals (*p* ≤ 0.044), whereas the expression of *crf-r2* expression was significantly lower than in control fish (*p* ≤ 0.006). In contrast to the telencephalon, the *pomc A* expression in the hypothalamus of female fish showed only significantly higher values in the feed reward and air exposure groups than in control fish (*p* ≤ 0.034).

The analyses of expression patterns of CRF-related genes in the optic tectum of male fish showed that *crf1* and *crf-r1* are significantly increased in the feed reward group compared with the controls (Figure 4, *p* ≤ 0.036). The *crf-r2* expression was also higher in the feed control group, but its mRNA levels were lower in the feed reward and air exposure groups than in control fish (*p* ≤ 0.044). Reduced gene expression was also noted for these treatment groups for the gene *uro1* in male fish. In addition, the *mr* transcript levels in the male optic tectum were higher in the feed reward and air exposure groups than in the controls (Figure 4, *p* ≤ 0.001). In female zebrafish, the expressions of *crf1* and *crf-r1* were lower in feed-rewarded and air-exposed fish (Figure 3, *p* ≤ 0.018), but the expression of both genes was significantly higher in feed control than in feed-rewarded fish (*p* = 0.024). The mRNA levels of *crf-r2* and *crh-bp* were significantly lower in all treatments than in the control animals in the female optic tectum (Figure 3, *p* ≤ 0.001). The expression of *pomc A* was not only significantly lower in all treatments than in the controls (*p* ≤ 0.001) but also lower in the feed reward group than in the feed control or air exposure groups (*p* ≤ 0.046). The *uro1* transcript levels were significantly higher in all treatments than in the controls (*p* ≤ 0.014). In addition, the expression of *gr* in female fish was significantly different between the feed reward group and air-exposed fish compared with the controls (*p* ≤ 0.026).

The HPI axis–related genes in the rhombencephalon showed that the expression of *crf-r2* in male fish was higher in the feed reward group than in the controls or the feed control group (Figure 4, *p* ≤ 0.04). The mRNA levels of *crh-bp* were found to be significantly higher in all treatment groups than in control fish (*p* ≤ 0.006) and also different between the feed-rewarded and air-exposed fish (*p* = 0.038). The expression of *pomc A* in the male rhombencephalon was lower in fish from the feed control group than the controls (*p* = 0.032). In the female rhombencephalon, the expression of *crf-r2*, *pomc A*, and *crh-bp* was significantly lower and the expression of *uro1* significantly higher in the feed reward and feed control groups than in control fish (Figure 3, *p* ≤ 0.048).

Taken together, the HPI axis–related genes were regulated differently in female and male fish, and the differences were observed in all four brain parts of the fish.

### 3.4 Gene expression patterns of appetite-related genes

Investigations of appetite-related gene expression patterns were also performed for all four different brain parts separately, and differences in the expression patterns in female and male fish were observed. Accordingly, the expression of *npy* was significantly higher in the feed reward group (Figure 4, *p* = 0.028) and fish exposed to air (*p* = 0.004) in the telencephalon of male fish.

In the hypothalamus of male fish, the mRNA levels of *cart* were found to be higher in the feed control and air exposure groups than in the controls (Figure 4, *p* < 0.004). In addition, the expression of *gal* was significantly higher in feed control fish than in feed-rewarded or air-exposed male fish (*p* < 0.01). In female fish, *cart* expression in the hypothalamus was found to be higher in all treatment groups than in the controls (Figure 3, *p* < 0.002). The same pattern was observed for the mRNA levels of *cck-a* in female fish (*p* ≤ 0.024). In addition, *gal* expression in the hypothalamus of female fish was significantly different in air-exposed animals than in the controls or feed-control fish (*p* ≤ 0.03).

The analyses of appetite-related genes in the optic tectum only resulted in a significant difference in the *npy* expression levels in male fish of the feed reward and feed control groups compared with control fish (Figure 4, *p* ≤ 0.038). In female fish, the expression of *cck-a* was found to be significantly higher in all treatments than in the control animals (Figure 3, *p* < 0.001), whereas the expression of *npy* and *gal* was significantly lower in all treatments than in the control (*p* < 0.001) and was also different between the feed reward group and feed control or air exposure group (*p* ≤ 0.02).

The appetite-related genes in the rhombencephalon of male fish showed that the expression of *agouti* is higher in the feed control animals than in control fish (Figure 4, *p* = 0.046). However, the *agouti* transcript levels in the rhombencephalon of female fish were also found to be lower in the feed reward and feed control groups than in control fish (Figure 3, *p* < 0.034). The expression of *cck-a* and *cck-b* was higher in the feed reward and feed control groups than in the controls (*p* ≤ 0.024), which was also observed for *cck-a* in the air exposure group (*p* = 0.020).

Overall, the investigation of the appetite-related genes identified significant differences between the experimental groups, which included differences between female and male fish. In addition, the effects of the stressors on appetite gene expression were not restricted to the hypothalamus.

### 3.5 Gene expression patterns of serotonergic and dopaminergic genes, metabolic genes, isotocin, and opioid receptors

The investigations of the expression patterns of the remaining genes belonging to a number of different pathways were also performed for all four different brain parts separately, and differences in the expression patterns between female and male fish were observed. In the telencephalon of male fish, the expression of *dopar 2a* was found to be significantly lower in air-exposed fish than in the controls (*p* = 0.02, Figure 4). The transcript levels of *iso pre* were found to be different for each treatment group (*p* < 0.001), and *iso-r2* expression differed between controls and feed-rewarded animals and air-exposed male zebrafish (*p* < 0.05). Female zebrafish showed that the expression of *iso pre* was only increased in the telencephalon of the feed control group, whereas the expression of its receptor *iso-r2* was significantly increased in feed-rewarded animals (Figure 3, *p* = 0.038). In the male telencephalon, the expression of *mTOR* was significantly higher in feed control than in feed-rewarded or air-exposed animals (*p* < 0.001). The *opio d1b* receptor only showed a higher expression in the feed control animals than in air-exposed male zebrafish (*p* = 0.048). Similarly, the expression of this gene was increased in female fish of the feed reward and feed control groups (Figure 3, *p* < 0.05). The transcript levels of the metabolic gene *pyrkin* were higher in control fish than in air-exposed male zebrafish (*p* = 0.032). By contrast, not *pyrkin*, but the metabolic gene *gapdh* was found to be differently expressed in the female telencephalon, with a significant lower expression value in the feed control group than in the controls or air-exposed fish (Figure 3, *p* < 0.038). The serotonin pathway–related gene *tph* in male fish showed a higher expression in feed-rewarded and feed control animals (*p* ≤ 0.032), whereas the expression of the *5 ht-r* (Figure 4) showed lower expression in feed-rewarded and air-exposed animals than in the control animals (*p* ≤ 0.006). Furthermore, the difference between the feed control and air-exposed animals was found to be significantly different (*p* = 0.048). In female zebrafish, the expression of *5 ht-r* was higher in feed control and air-exposed animals than in control or feed-rewarded fish (Figure 3, *p* ≤ 0.022). The *tph* mRNA levels in female fish were higher in all treatment groups than in the controls (Figure 3, *p* ≤ 0.01).

The same gene set in the hypothalamus showed that the mRNA expression of *iso pre* and *iso-r2* was significantly lower in all male treatment groups than in the controls (Figure 4, *p* < 0.004). Similar to the telencephalon, in the hypothalamus, the expression of *mTOR* was significantly higher in the male feed control animals than in feed-rewarded or air-exposed animals (*p* < 0.001). Furthermore, again, the *opio d1b* receptor showed the highest expression in the feed control animals in male zebrafish. In addition, the transcript levels of the metabolic gene *pyrkin* in male fish were higher in feed control fish than in control fish and feed-rewarded zebrafish (*p* < 0.010). The mRNA expression of *gapdh* was also the highest in the feed control group in male animals, with a significant difference between the controls (*p* < 0.001) and feed-rewarded animals (*p* = 0.032). In male zebrafish, *5 ht trb* was found to be significantly increased in fish belonging to the feed control group compared with the controls or air-exposed animals (Figure 4, *p* ≤ 0.032). A significant difference between these treatment groups was observed for *5 ht-r* in the same animals (*p* ≤ 0.044). The female zebrafish showed significantly lower *dopar 2a* transcript levels in the hypothalamus of fish in all treatment groups than the controls (Figure 3, *p* ≤ 0.028). The mRNA expression of *iso pre* in the female hypothalamus was significantly higher in feed control fish and fish exposed to air than in the controls (Figure 3, *p* < 0.02). In addition, the expression of *iso-r1* in the female hypothalamus was significantly higher in all treatments than in the controls (Figure 3, *p* ≤ 0.006), and the expression of *iso-r2* was only significantly higher in air-exposed fish than in the controls (*p* = 0.018). In the female hypothalamus, the mRNA expression of *5 ht-r* was also found to be higher in the feed reward and air exposure groups than in the controls (Figure 3, *p* ≤ 0.022).

The analyses for the optic tectum showed that the *iso-r2* mRNA expression in male fish was significantly lower in all treatments than in the controls (Figure 4, *p* ≤ 0.028). The serotonin pathway–related gene *tph* in male fish showed a lower expression in feed-rewarded and air-exposed animals (*p* ≤ 0.02), whereas *5 ht-r* showed different expression levels between all three treatments (*p* ≤ 0.04). Moreover, the *th2* transcript levels in the optic tectum of male fish were significantly different between all treatment groups and controls (*p* ≤ 0.008) and also between the feed-rewarded fish and air-exposed animals (*p* = 0.014). Similar to the observations for the hypothalamus in female fish, the expression of *dopar 2a* and *mTOR* was significantly lower in the optic tectum in all female treatment groups than in the controls (Figure 3, *p* ≤ 0.01). The expression of *citrsyn* and *pyrkin* in female fish was significantly higher in all treatment groups than in control fish (*p* ≤ 0.036), whereas the mRNA levels of *succdh* were higher only in the feed reward group than in the controls (*p* = 0.018). In addition, the expression of *opio d1b* was higher only in the feed reward group than in the controls (*p* = 0.036). The *iso-r1* transcript levels were higher in all treatment groups than in the control animals (*p* ≤ 0.006), but the expression of *iso-r2* was found to be different between the feed reward and air exposure groups compared with the controls (*p* ≤ 0.026). In addition, the expression of *iso-r2* was different between female fish from the feed reward group and feed control group (*p* = 0.04). Finally, the *th1* mRNA levels were significantly different in the optic tectum of female fish between the feed reward and feed control groups compared with the controls (*p* ≤ 0.042) but only significant for *th2* between the feed reward group and control fish (*p* ≤ 0.006).

For the same gene set in the male rhombencephalon, it was noted that the expression of *dopar 2a* was found to be significantly lower in air-exposed and feed-rewarded fish than in the controls (Figure 4, *p* ≤ 0.04). The expression of *gapdh* was higher in the feed reward group, and the expression of *succdh* was significantly lower than in the controls (*p* ≤ 0.004). The expression of *mTOR* was high in the feed control group compared with fish of the other groups (*p* < 0.001). Similarly, mRNA levels of *opio d1a* and *opio d1b* were significantly higher in the feed control group than in the controls (*p* ≤ 0.038). In addition, the expression of *iso pre* was lower in the feed control group than in the remaining groups in the male rhombencephalon (*p* ≤ 0.001), whereas the transcript levels of *iso-r2* were significantly lower in all treatment groups than in the controls (*p* ≤ 0.028). Moreover, the expression of *tph* in the male rhombencephalon was significantly lower in the feed reward group than in the controls or feed controls (*p* ≤ 0.044). Finally, the expression of the *5 ht-r* was significantly lower in the feed reward group than in control fish (*p* = 0.044). By contrast, the mRNA expression of *gapdh* and *citrsyn* in the rhombencephalon of female fish showed a higher transcript level in the feed reward and feed control groups than in the zebrafish of the control group (*p* ≤ 0.032). The expression of *opio d1b* in female fish was significantly higher in the feed-rewarded animals than in the controls (*p* = 0.038). In addition, female fish showed a lower expression of *mTOR* in air-exposed fish than in the other groups (Figure 3, *p* ≤ 0.042). Furthermore, the expression of *iso pre* was lower in the feed reward and feed control groups (*p* ≤ 0.002), but the expression of *iso-r1* was higher in all treatments than in control fish (*p* ≤ 0.036). Finally, the transcript levels of *th1* in female fish were found to be higher in the feed reward and feed control groups than in the controls (*p* ≤ 0.034), and the expression of the *5 ht-r* was only higher in the air-exposed animals than in control fish (*p* = 0.016).

### 3.6 Principal component analyses

**FIGURE 5:**
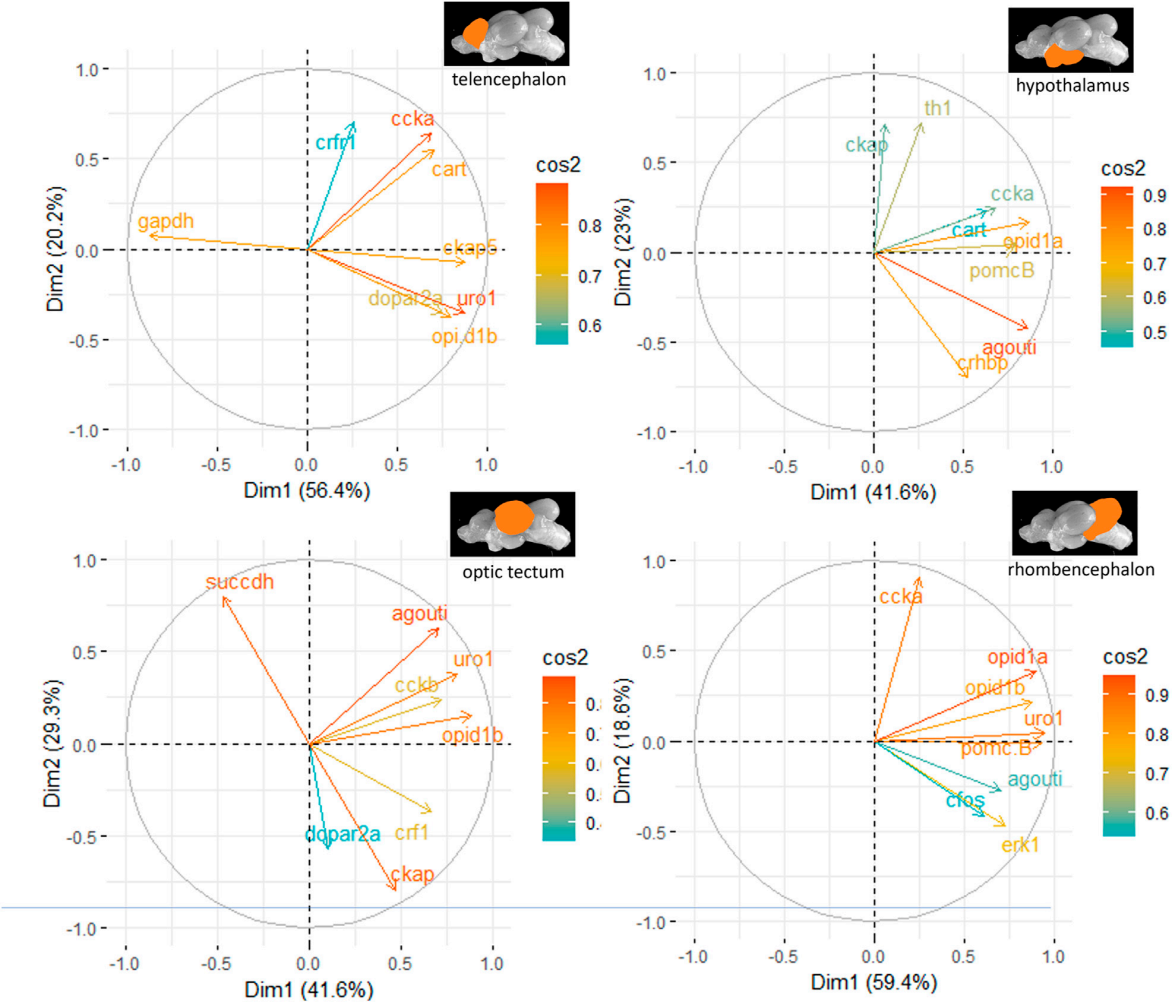
Gene expression analysis with PCAs for the most contributing genes in each of the four brain parts showing their representation on the factor map as cos^2^ values (i.e., high cos^2^ values indicate high influence on the gene expression patterns), where the numbers on the *x*-axis (Dim1) and *y*-axis (Dim2) indicate the percentage of variance in the datasets that is explained by the first two components of the PCA of female fish; n = 6 per treatment.

**FIGURE 6:**
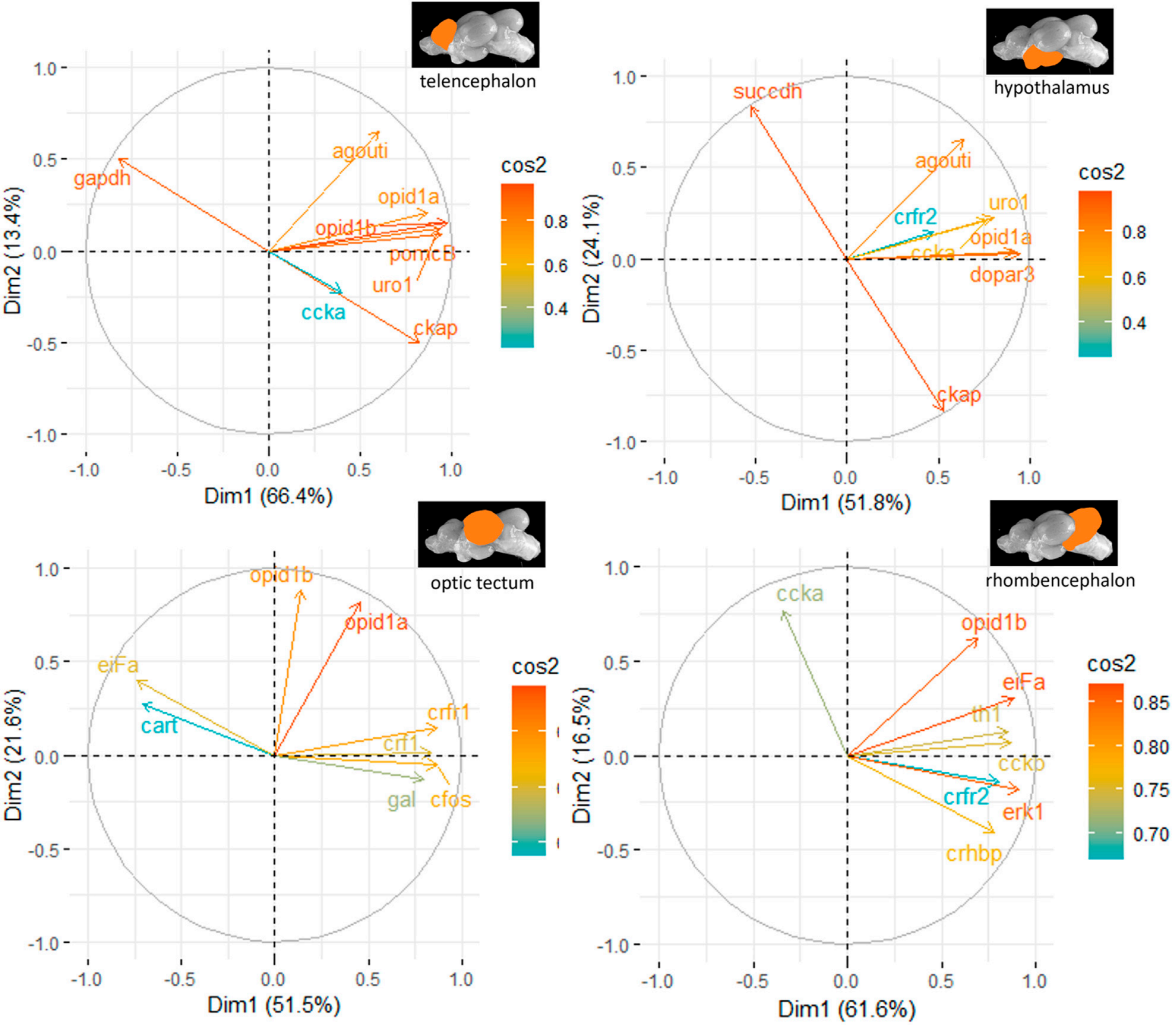
Gene expression analysis with PCAs for the most contributing genes in each of the four brain parts showing their representation on the factor map as cos^2^ values (i.e., high cos^2^ values indicate high influence on the gene expression patterns), where the numbers next to the *x*-axis (Dim1) and *y*-axis (Dim2) indicate the percentage of variance in the data sets that is explained by the first two components of the PCA of male fish; n = 6 per treatment.

The principal component analyses for all genes and brain parts are shown in Supplementary Figures S2–S5. Based on these results, the two most important genes for each brain regulation pathway have been selected and used for additional PCA. For the genes in the telencephalon of female fish, *cck-a* and *uro1* were identified as genes with strong relevance for the expression patterns in this brain part (Figure 5). By contrast, the contribution of *cck-a* to the outcome of gene expressions in the male telencephalon was considerably low compared with the contribution of genes such as *opio d1b* and *gapdh* (Figure 6). Moreover, the genes with the highest influence in the hypothalamus of female fish were *agouti* and *opio d1a*, which were also found in the top eight of the list for male hypothalamus, but in the latter, the influence of *succdh*, *ckap*, and *dopar3* was even higher than that. In the optic tectum of female fish, the genes *agouti* and *ckap* had the strongest ability to explain the variability in the data sets, whereas in the male optic tectum, this was observed for the two opioid receptors (Figure 6). Finally, the female rhombencephalon showed the strongest influence of the genes *opio d1a* and *uro1*, whereas *eiFa* and *opio d1b* were influential in the male rhombencephalon.

### 3.7 Classification and feature importance

The random forest classification reached 100% accuracy for the genes selected for the test data set in the telencephalon of female and male fish but only 78% accuracy for the genes in the male hypothalamus. The latter result was caused by lower classification success for the treatment in the feed group and in the feed control group (Table 1).

**TABLE 1:** Results of the precision, recall, and F1 scores for the random forest models for the test data sets of each brain part separately for female and male fish; models based on the bootstrapped gene expression data sets; n = 100 per treatment group.

Brain part/gender	Treatment group	Model performance: precision, recall, F1 score
Telencephalon/male fish	Contr	1.00, 1.00, 1.00
	Feed	1.00, 1.00, 1.00
	Feed contr	1.00, 1.00, 1.00
	Air exposure	1.00, 1.00, 1.00
Hypothalamus/male fish	Contr	0.56, 0.50, 0.53
	Feed	0.55, 0.60, 0.57
	Feed contr	1.00, 1.00, 1.00
	Air exposure	1.00, 1.00, 1.00
Optic tectum/male fish	Contr	1.00, 1.00, 1.00
	Feed	1.00, 1.00, 1.00
	Feed contr	1.00, 1.00, 1.00
	Air exposure	1.00, 1.00, 1.00
Rhombencephalon/male fish	Contr	1.00, 1.00, 1.00
	Feed	1.00, 1.00, 1.00
	Feed contr	1.00, 1.00, 1.00
	Air exposure	1.00, 1.00, 1.00
Telencephalon/female fish	Contr	1.00, 1.00, 1.00
	Feed	1.00, 1.00, 1.00
	Feed contr	1.00, 1.00, 1.00
	Air exposure	1.00, 1.00, 1.00
Hypothalamus/female fish	Contr	1.00, 1.00, 1.00
	Feed	1.00, 1.00, 1.00
	Feed contr	1.00, 1.00, 1.00
	Air exposure	1.00, 1.00, 1.00
Optic tectum/female fish	Contr	1.00, 1.00, 1.00
	Feed	1.00, 1.00, 1.00
	Feed contr	1.00, 1.00, 1.00
	Air exposure	1.00, 1.00, 1.00
Rhombencephalon/female fish	Contr	1.00, 1.00, 1.00
	Feed	1.00, 1.00, 1.00
	Feed contr	1.00, 1.00, 1.00
	Air exposure	1.00, 1.00, 1.00

The classification of genes in the telencephalon of male fish with random forest models showed that the entire data set of 39 genes resulted in 100% accuracy for the classification (Table 2). According to the feature importance for gene expression patterns of this brain part, the top ten genes from this list together contributed to 71.22% of the importance for the model (Table 3), and two genes (*iso pre* and *crf-r2*) were sufficient to maintain the accuracy of the random forest model (Table 2). Similarly, of the 39 genes in the female telencephalon in total, the top ten genes from the feature importance list made up 66.9% of the importance for the model, and nine genes were sufficient to achieve 100% accuracy of the classification. For the hypothalamus of male fish, the top ten genes from this list together contributed to 60.22% of the importance for the model, whereas the same calculation for the female hypothalamus accounted for 65.85% of the importance for the model (Table 3). Similarly, the top ten genes from this list together contributed to 62.04% of the importance for the model for genes in the optic tectum of male fish, whereas the same calculation for the female hypothalamus accounted for 69.48% of the importance for the model (Table 3). For the rhombencephalon of male fish, the top ten genes from this list together contributed to 64.36% of the importance for the model, whereas the same calculation for the female hypothalamus accounted for 68.92% of the importance for the model (Table 3). The feature lists for each brain part commonly consist of HPI axis–related and serotonergic genes (highlighted in Table 3).

**TABLE 2:** Results of the random forest models for each brain part separately for female and male fish. The accuracy of the model achieved by including all available gene expression data was compared with the minimum number of genes to calculate the same accuracy of the model; models based on the bootstrapped gene expression data sets; n = 100 per treatment group.

Brain part/gender	Maximum no. of genes	% Accuracy if all genes are included	Minimum no. of genes for 100% accuracy (as listed in the feature importance in Table 2)
Telencephalon/male fish	39	100	2
Hypothalamus/male fish	40	78	-
Optic tectum/male fish	43	100	7
Rhombencephalon/male fish	36	100	7
Telencephalon/female fish	39	100	9
Hypothalamus/female fish	40	100	7
Optic tectum/female fish	43	100	5
Rhombencephalon/female fish	36	100	6

**TABLE 3:** Feature importance of the random forest models for each brain part separately for the full range of genes available for female and male fish; models based on the bootstrapped gene expression data sets; n = 100 per treatment group; HPI axis–related genes are shaded in blue, serotonergic genes are shaded in green, and isotocin-related genes are shaded in yellow.

Brain part/gender	Feature importance of top ten (% contribution to the model)									
Telencephalon/male fish	*iso pre*	*crf-r2* (11.07)	*crf-r1* (9.92)	*5 ht-r* (8.41)	*mTOR* (8.39)	*palld* (5.64)	*gr* (4.52)	*iso-r2* (3.43)	*ckap5* (2.39)	*Dopar 2a* (2.38)
	(15.07)									
Hypothalamus/male fish	*crf-r2* (10.0)	*iso-r2* (8.84)	*5 ht-r* (6.88)	*iso pre* (6.68)	*ef* (6.47)	*crf-r1* (6.02)	*eiFa* (4.69)	*pyrkin* (3.56)	*tub* (3.59)	*cck-a* (3.59)
Optic tectum/male fish	*5 ht-r* (14.96)	*mr* (6.97)	*th1* (6.48)	*crf-r2* (6.08)	*5 ht* *tr* *a*	*dopar3* (5.41)	*opio d1b* (4.65)	*iso-r2* (4.10)	*crf-r1* (3.96)	*opio d1a* (3.90)
					(5.53)					
Rhombencephalon	*erk-1*	*crf-r2*	*mTOR*	*iso-r2*	*pomc B*	*dopar 2a* (5.60)	*crh-bp* (5.29)	*palld* (5.24)	*5 ht-r*	*eiFa*
/male fish	(11.50)	(8.11)	(7.35)	(6.02)	(5.67)				(4.84)	(4.74)
Telencephalon/	*5 ht-r*	*tph*	*opio d1b*	*ckap5*	*uro1*	*eiFa*	*succdh*	*mr*	*cck-b*	*gapdh*
Female fish	(13.17)	(11.82)	(8.81)	(6.36)	(5.80)	(5.40)	(4.05)	(4.04)	(3.88)	(3.57)
Hypothalamus/	*crf-r2*	*5th* *tr* *b*	*iso-r2*	*gal*	*iso pre*	*ckap5*	*mTOR*	*dopar 2a*	*cart*	*crf-r1*
Female fish	(11.07)	(8.01)	(7.99)	(7.35)	(6.43)	(6.15)	(5.18)	(4.86)	(4.71)	(4.10)
Optic tectum/	*pomc A*	*crf-r2*	*tub*	*gal*	*18S RNA*	*npy*	*iso-r2*	*dopar 2a*	*th1*	*crf1*
Female fish	(14.14)	(9.58)	(8.08)	(7.64)	(7.35)	(5.21)	(4.88)	(4.42)	(4.19)	(3.99)
Rhombencephalon/female fish	*iso pre* (9.94)	*crf-r2* (8.83)	*egr-1* (8.44)	*dopar3* (6.95)	*iso-r1* (6.85)	*erk-1* (6.42)	*citrsyn* (5.91)	*mr* (5.60)	*5 ht-r* (5.09)	*mTOR* (4.89)

## 4 Discussion

### 4.1 Morphological characteristics of the fish and stress hormone levels

The female and male fish showed normal variation in their morphological characteristics between treatment groups (Supplementary Figure S1). Furthermore, the observed gender-specific differences in weight for adult zebrafish are typical for this species (Green et al., 2023). In the course of the present study, it was confirmed that there are only morphological differences between female and male fish. Accordingly, there were also gender-specific effects on the whole-body stress hormone concentration, although the sampling point of 60 min after stress application may not have been optimal to provide detailed evidence for this. Air exposure or an increase in water current is known to result in a rapid increase in whole-body cortisol levels (Barcellos et al., 2007; Ramsay et al., 2009; Fuzzen et al., 2010; Pavlidis et al., 2013). For example, Pavlidis et al. (2015) reported trunk cortisol levels that started at approximately 15 min and returned to basal levels at approximately 2 h following exposure to acute stressors. For net-handled zebrafish, the whole-body cortisol level was similar to the control cortisol level already at 60 min after the treatment (Ramsay et al., 2009). This was probably the reason why there was no significant difference in the body cortisol and cortisone levels detectable at 60 min after the treatment in the present study. Still, the clear correlation between the cortisol and cortisone whole-body levels indicates that fish with a higher body cortisol level also show higher cortisone levels, whereby the latter indicates a higher turnover of cortisol. However, stress hormone levels in fish are affected by many regulatory pathways and environmental influences (Barton, 2002), making the interpretation of body cortisol levels rather difficult.

### 4.2 Gene expression analyses

Increased expression of *c-fos* in the telencephalon of distressed fish is commonly accepted as a suitable indicator of brain activity in fish species such as zebrafish, goldfish *Carassius auratus*, and common carp *Cyprinus carpio* (Fujikawa et al., 2006; Lau et al., 2011; Pavlidis et al., 2015; Burren and Pietsch, 2021). However, the present, broader study on brain gene expression indicates that other genes are more appropriate to indicate the influence of different stressors on zebrafish, for example, the opiod receptors or the expression of the metabolic gene *gapdh.* Moreover, the genes with the strongest congruency with the treatments appear to be different for female and male zebrafish.

Similar results have been observed for the appetite genes that have been included in the present study. The effects of stress on food intake of fish are possible due to the connection of NPY and CRF neurons (Bernier et al., 2004; Kohno et al., 2016). In goldfish forebrains, cortisol administration resulted in a dose-dependent increase in *npy* mRNA (Bernier et al., 2004). Handling stress also caused increased *npy* mRNA expression in the hypothalamus of zebrafish (Cortés et al., 2018). Similar results have been observed for crowding stress in rainbow trout, *O. mykiss*, but not in tilapia, *O. mossambicus* (Conde-Sieira et al., 2010; Upton and Riley, 2013; Naderi et al., 2018). In addition, other brain regions in fish also exhibit different gene expression patterns due to exposure to stress. For instance, intra-peritoneal administration of cortisol in tilapia resulted in decreased *npy* mRNA expression in the telencephalon but not in the hypothalamus (Janzen et al., 2012). Anorexigenic neuropeptides, such as *cck* and *cart*, were found to be less expressed in the animals exposed to stressors, which confirms their appetite downregulating function upon stress; however, the effects have been less pronounced in carp at 1 hour after being exposed to similar stressors (Pawlak et al., 2023). The expression of *gal* was low in air-exposed female fish and high in male zebrafish belonging to the feed control group, which confirms the central role of GAL as a feeding inducing neuropeptide (Zhang et al., 2023) that is expressed at high levels when fish are hungry or reduced upon stress. The expression of AGOUTI signaling proteins in zebrafish has already been related to the regulation of feeding behavior and modulation of the stress axis *via pomc A* (Shainer et al., 2019). In the present study, *agouti* mRNA expression was only significantly different in the rhombencephalon of zebrafish, with female fish showing different effects of the treatments than male fish. Nevertheless, this indicates that not only the AGOUTI signaling proteins, as suggested by Shainer et al. (2019), but also AGOUTI plays a central role in the regulation of the stress axis in zebrafish. However, in this respect, it should also be noted that feeding status is known to influence stimulus processing, for example, in the zebrafish tectum (Filosa et al., 2016). Thus, the feeding regime of the present study certainly also contributed to the outcome of the investigations of gene regulations in the brain.

The different stressors that have been used in the present study all resulted in responses from HPI axis–related genes. The regulation of *crf-r1* and *crf-r2* differed from the regulation patterns that were observed for similarly stressed carp (Pawlak et al., 2022). Faught and Vijayan (2022) assumed that a response of CRH-R1 leading to an increase in stress hormone levels is required for initiating and maintaining the behavior observed in acutely stressed zebrafish. In addition, the authors showed evidence for an important role of *mr* in prolonged stress response in zebrafish (Faught and Vijayan, 2018; 2022). The importance of *mr* is also emphasized by the fact that a loss of *gr* is not lethal in juvenile zebrafish (Griffiths et al., 2012). The expression of *gr* was not significantly different in fish of the present study except for its high expression in the optic tectum of female fish in the feed group and air exposure group, while the *mr* expression was higher in the optic tectum of fish from the same groups of male fish.

In addition, the expression of *pomc A* was assumed to be a readout of the GR function in zebrafish. *Pomc A* is increased by CRH stimulation (Alsop and Vijayan, 2008) and negatively regulated when increased body cortisol levels activate GR, which is a part of the HPI axis feedback loop (Griffiths et al., 2012; Ziv et al., 2014). In most brain parts, reduced *pomc A* expression in the stressed animals was observed, which may have been caused by previous short-lived cortisol increases. Glucocorticoid signaling *via* GR and MR and the hypoxia pathways appear to be connected in zebrafish, which may be especially relevant for the air exposure group in which stress responses due to handling and potential hypoxia due to lifting the fish out of the water occur at the same time (Marchi et al., 2020). Hypoxia-inducible factor (HIF) transcription factors have not been measured in the present study, but it can be speculated that the downregulation of *pomc A* in almost all brain parts was at least partly mediated by HIF. In addition, *tph* expression, considered to be a specific readout for the activity of serotonergic neurons in fish (Chivite et al., 2021), was found to be expressed higher in the telencephalon of stressed female and male fish, which indicates a general contribution of serotonergic pathways to stress responses.

### 4.3 Gene expression patterns observed by unsupervised learning methods

The variation in the data sets that could be explained by the first two dimensions in the PCA showed satisfactory results, with a mean value of 72.5% for the female brain parts and a slightly higher average value of 76.7% for the male brain parts. Furthermore, the PCA results showed a number of genes that had a stronger influence on the gene expression patterns for each treatment group than others. For example, the opioid receptors are among the genes with a high influence on the expression patterns when PCA is used, which further emphasizes the possible occurrence of stress-induced analgesia in fish (Thomson et al., 2020). However, despite the opioid receptors, there were substantial gender-specific differences between the top eight genes, mainly contributing to the outcome of the gene expressions. This results in the assumption that, so far, no genes could be identified that indicate the type of stress if unsupervised learning methods, such as a PCA, are employed. It could not, however, be excluded that the time point at 60 min after stress application also had a certain influence on this result. Therefore, additional experiments would be required to evaluate the optimal time point for investigating gene expression patterns after stress application. Since habituation to stocking densities and tank sizes are known to influence the behavior of zebrafish (Shishis et al., 2022), additional experiments should be conducted under comparable environmental conditions to allow comparison of the results.

### 4.4 Classification accuracy and feature importance

The classification models revealed that at least two genes in the top ten feature importance lists belonged to HPI axis–related genes. Hence, all the different stressors involved responses to HPI axis–related genes. Increased cortisol leads to the downregulation of *gr*, which downregulates the HPI axis in negative feedback. Serotonin is proposed to upregulate *gr* expression and possibly also *mr* expression (Griffiths et al., 2012) and thus can restore normal HPI responsiveness. Consequently, it is not surprising that HPI axis–related and serotonergic genes are most common in the top ten feature lists for the random forest models for each brain part. In addition, genes related to the isotocin pathway were commonly found in the top ten feature importance lists. Isotocin signaling through its receptors in the zebrafish is known to affect social behavior (Landin et al., 2020). Although the selection of small rearing groups in the present study may have influenced the social context of the zebrafish in general, it is assumed that low stocking densities often do not significantly affect their stress responses (Andersson and Kettunen, 2021). Hence, the influence of the applied stressors on isotocin signaling in the present study indicates further involvement of social parameters in stress responses. Nevertheless, more studies would be required to further understand the gender-specific differences in stress responses.

## 5 Conclusion

The applied stressors differently regulate genes in the zebrafish brain. A central role of the HPI axis–related genes, serotonergic genes, and isotocin-related genes was confirmed. A study using additional stressors for comparison is currently ongoing and will probably yield more detailed insights into gene regulation in zebrafish.

## Data Availability

The data sets presented in this study can be found in online repositories. The names of the repository/repositories and accession number(s) can be found at https://figshare.com/articles/dataset/PCR_files_and_fish_sampling_data/24942720.
