# Supplementary material for: Multiple faces of stress in the zebrafish (Danio rerio) brain
Source: Front Physiol. 2024 Apr 15;15:1373234. doi: 10.3389/fphys.2024.1373234 (PMC11070943; doi:10.3389/fphys.2024.1373234)
Supplement: Supplementary file 1 [file DataSheet1.docx]

Supplementary Material

## Supplementary Figures

females males


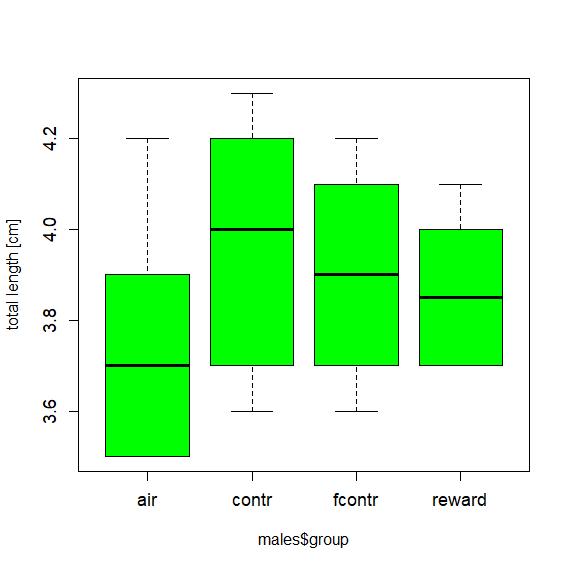

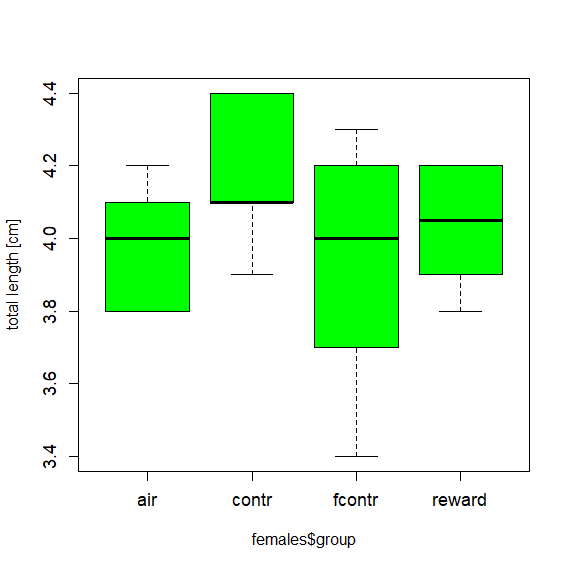


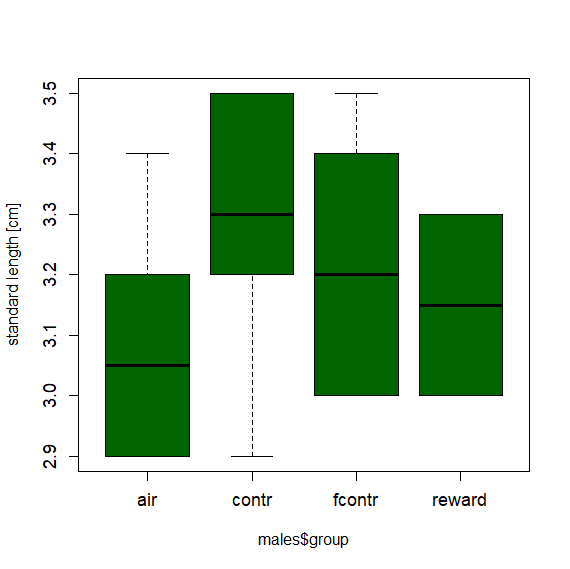

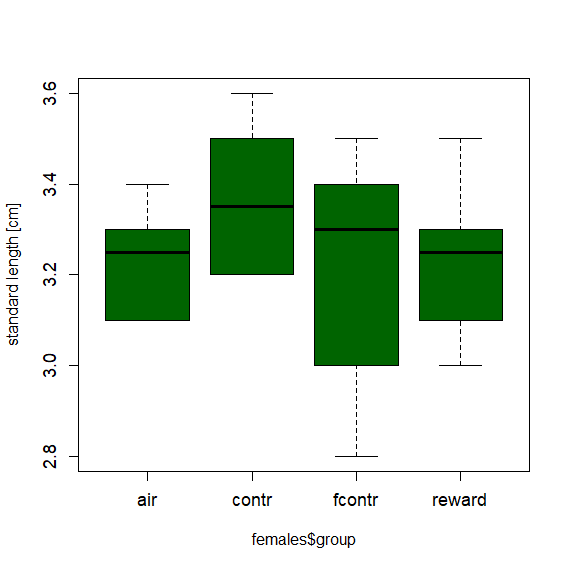


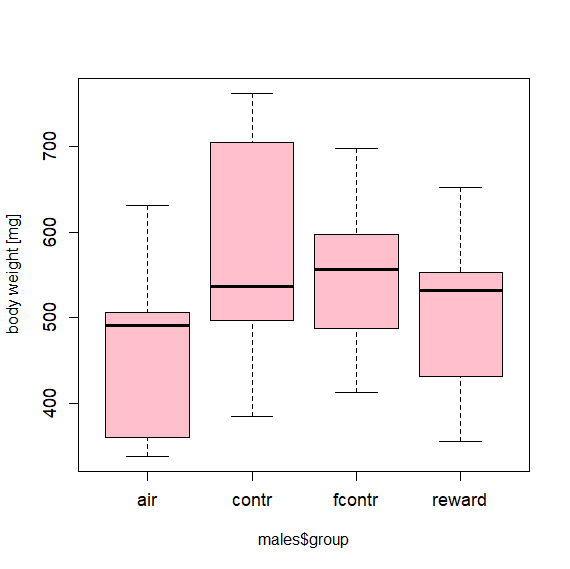

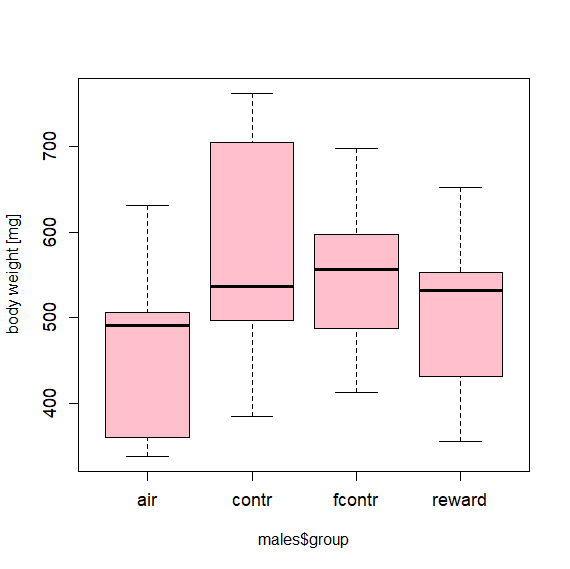


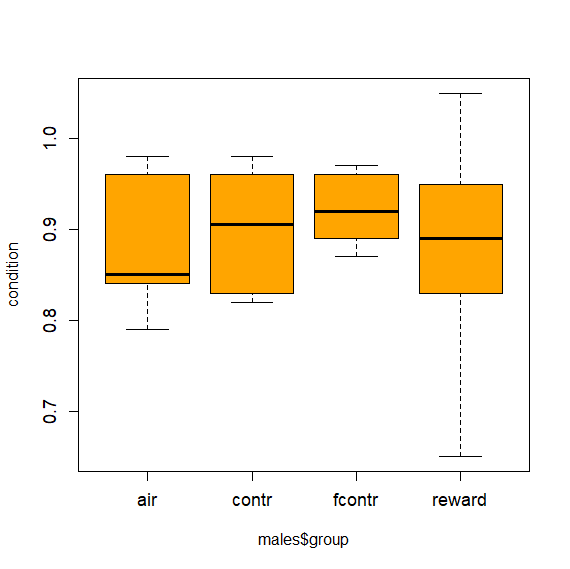

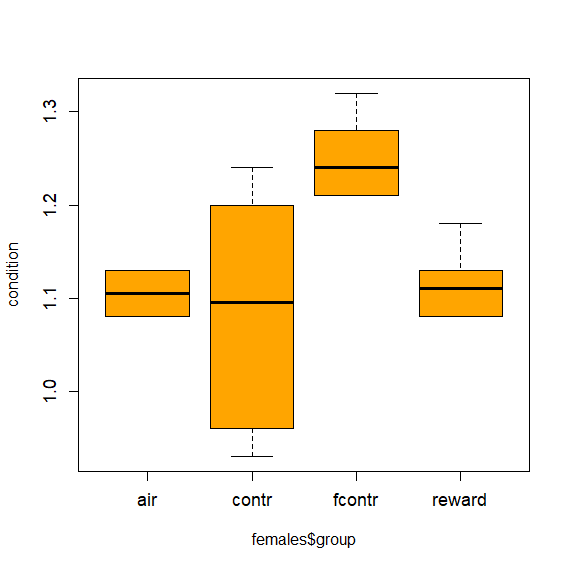


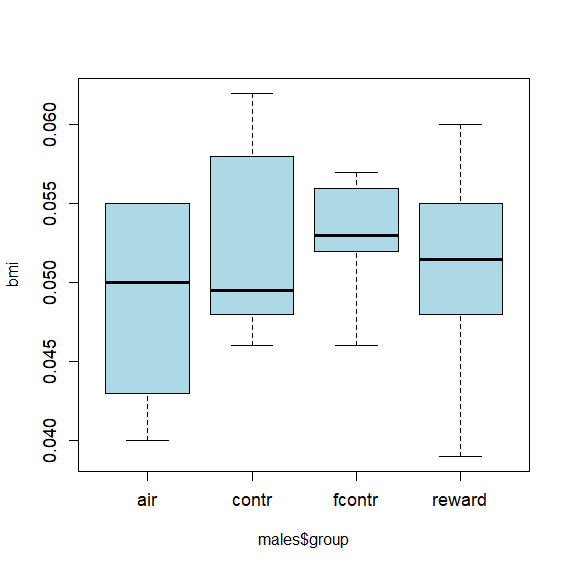

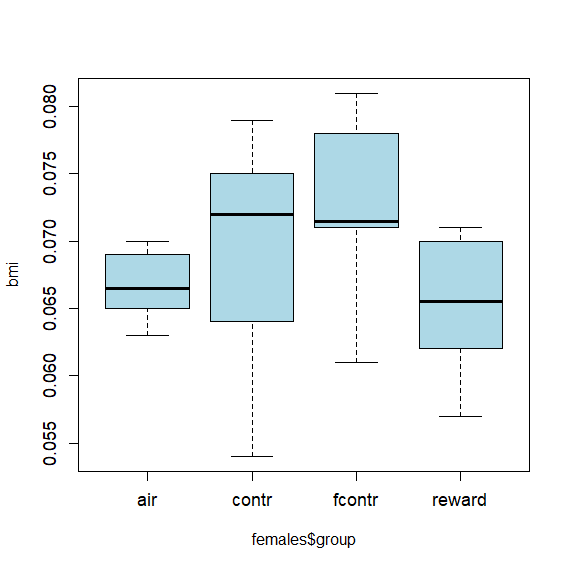


**Supplementary Figure S1.** Morphological differences between females and males for the treatment groups exposed to air (air), control fish (contr), fish without feeding (fcontr) or fish fed in the morning (reward); TL = total body length, SL = standard body length, condition = Fulton`s condition factor, bmi = body mass index, n = 6 for each group.

Females

**
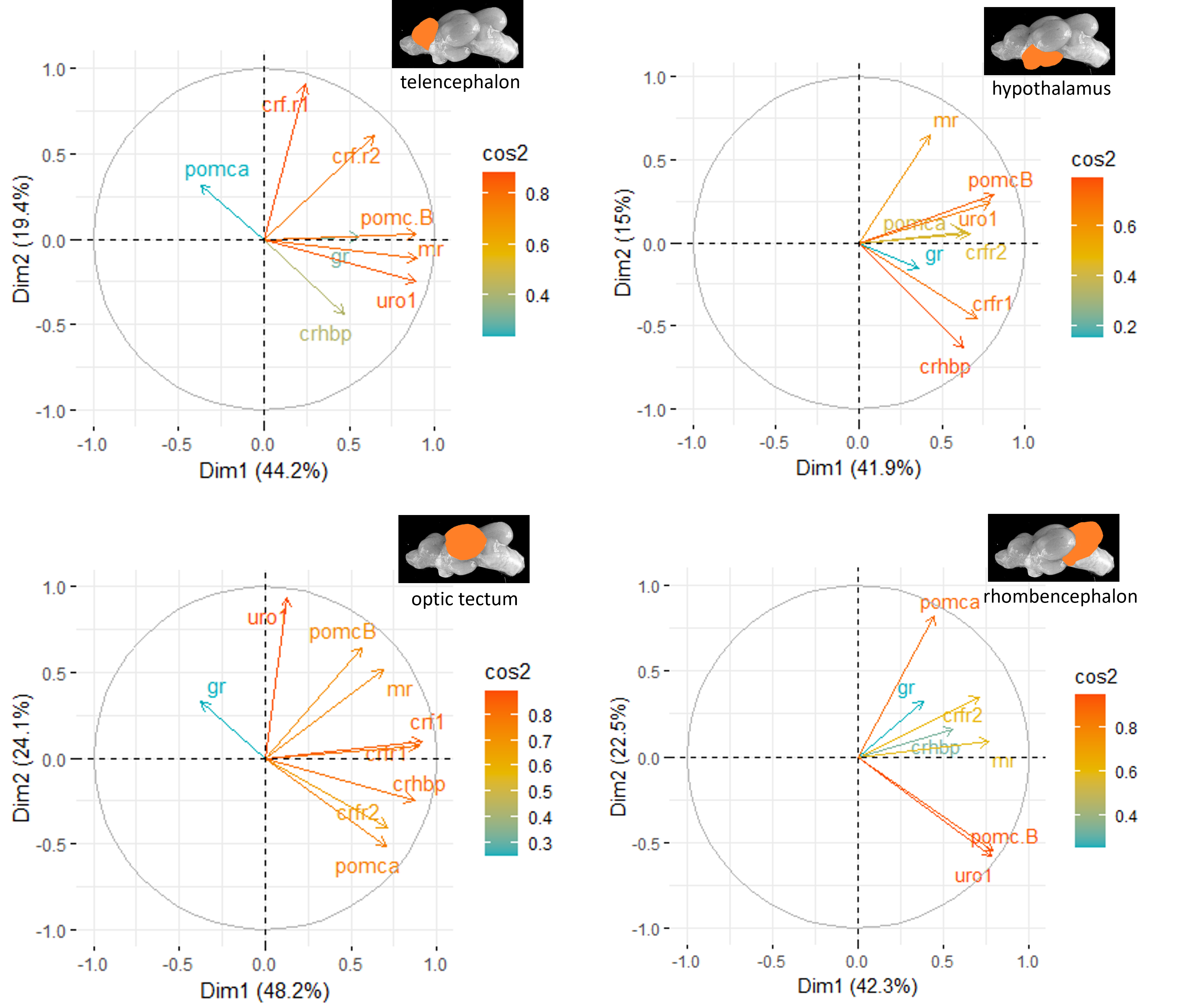
**

Males

**
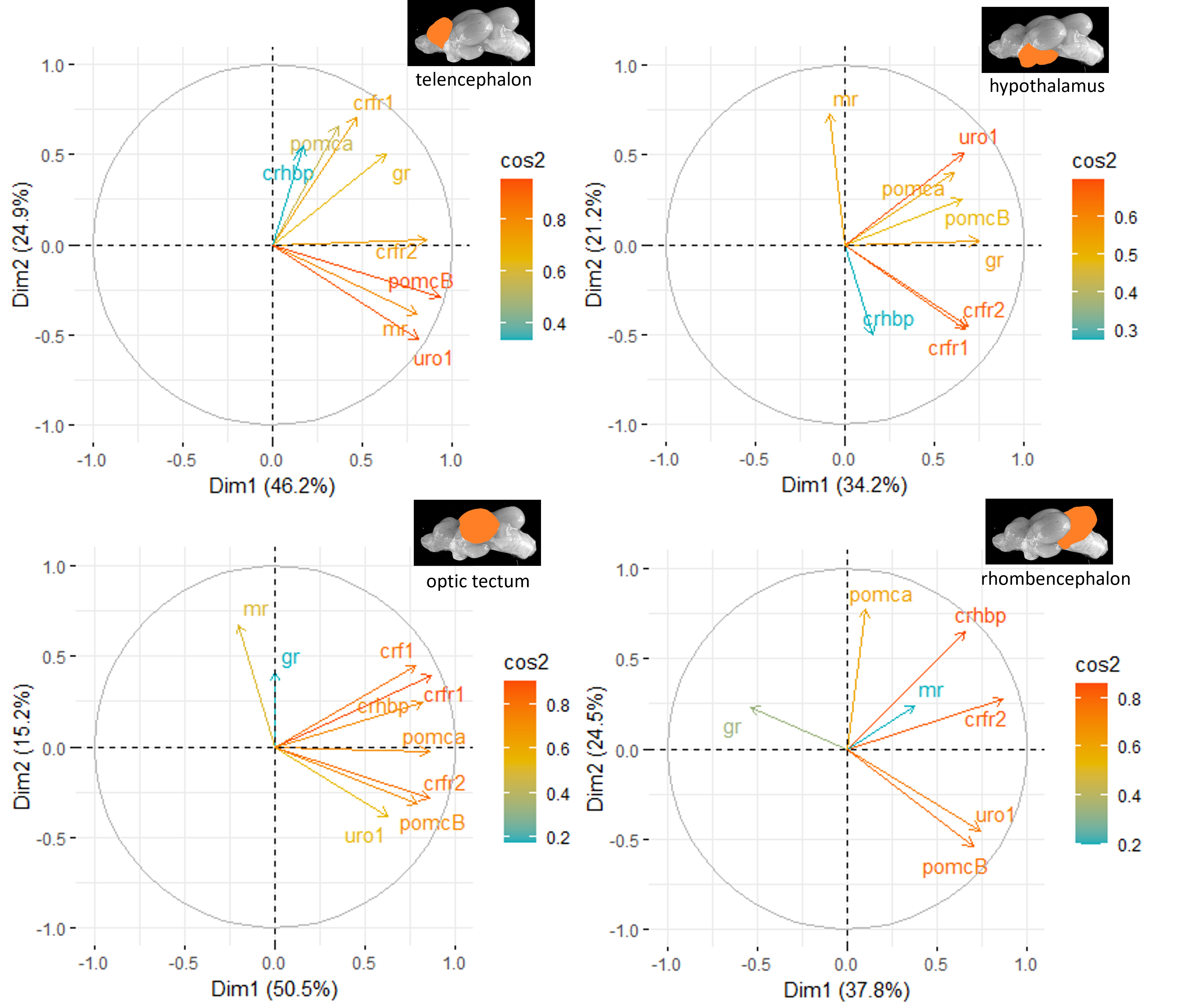
**

**Figure S2.** Gene expression analysis with Principal Component Analyses (PCA) for the contributing genes to the HPI pathway in each of the 4 brain parts showing their representation on the factor map as cos2 values, whereby the numbers next to Dim1 and Dim2 indicate the percentage of the variance in the data sets that is explained by the first two components of the PCA of female (upper 4 figures) and male fish (lower 4 figures); n = 6 per treatment.

Females

**
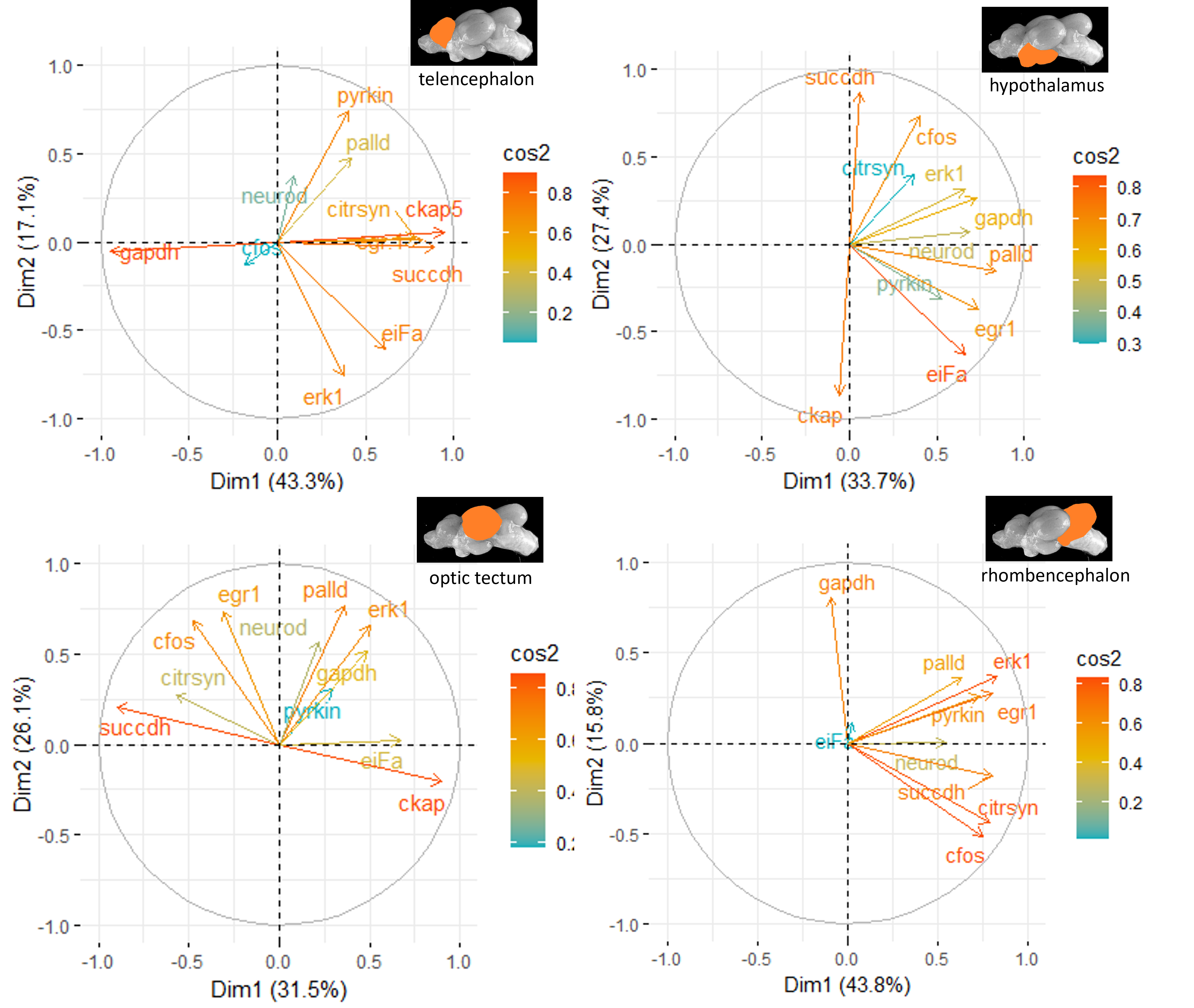
**

Males

**
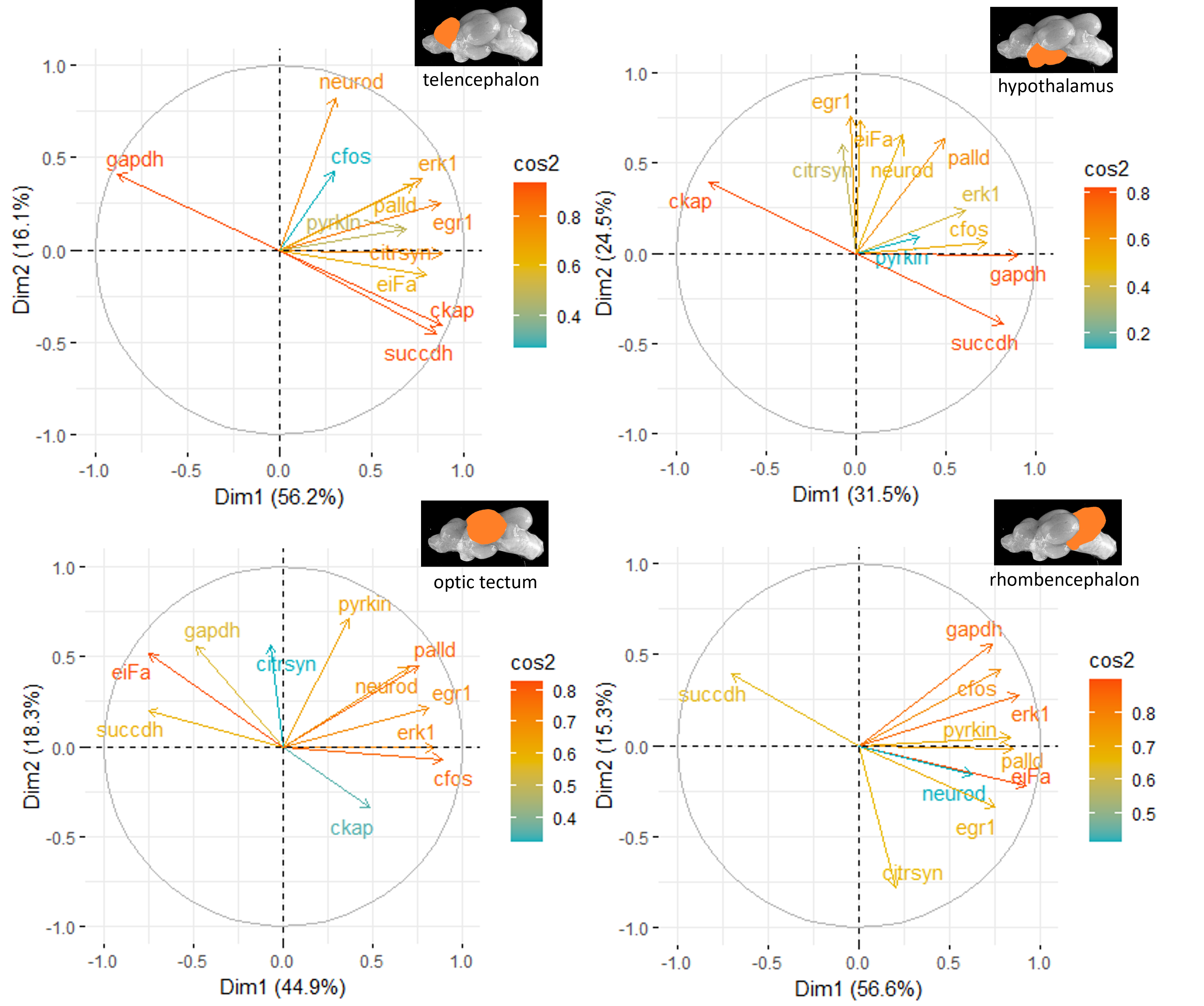
**

**Figure S3.** Gene expression analysis with Principal Component Analyses (PCA) for the contributing genes belonging to the immediate early genes (IEGs) and metabolic genes in each of the 4 brain parts

showing their representation on the factor map as cos2 values, whereby the numbers next to Dim1 and Dim2 indicate the percentage of the variance in the data sets that is explained by the first two

components of the PCA of female (upper 4 figures) and male fish (lower 4 figures); n = 6 per treatment.

Females


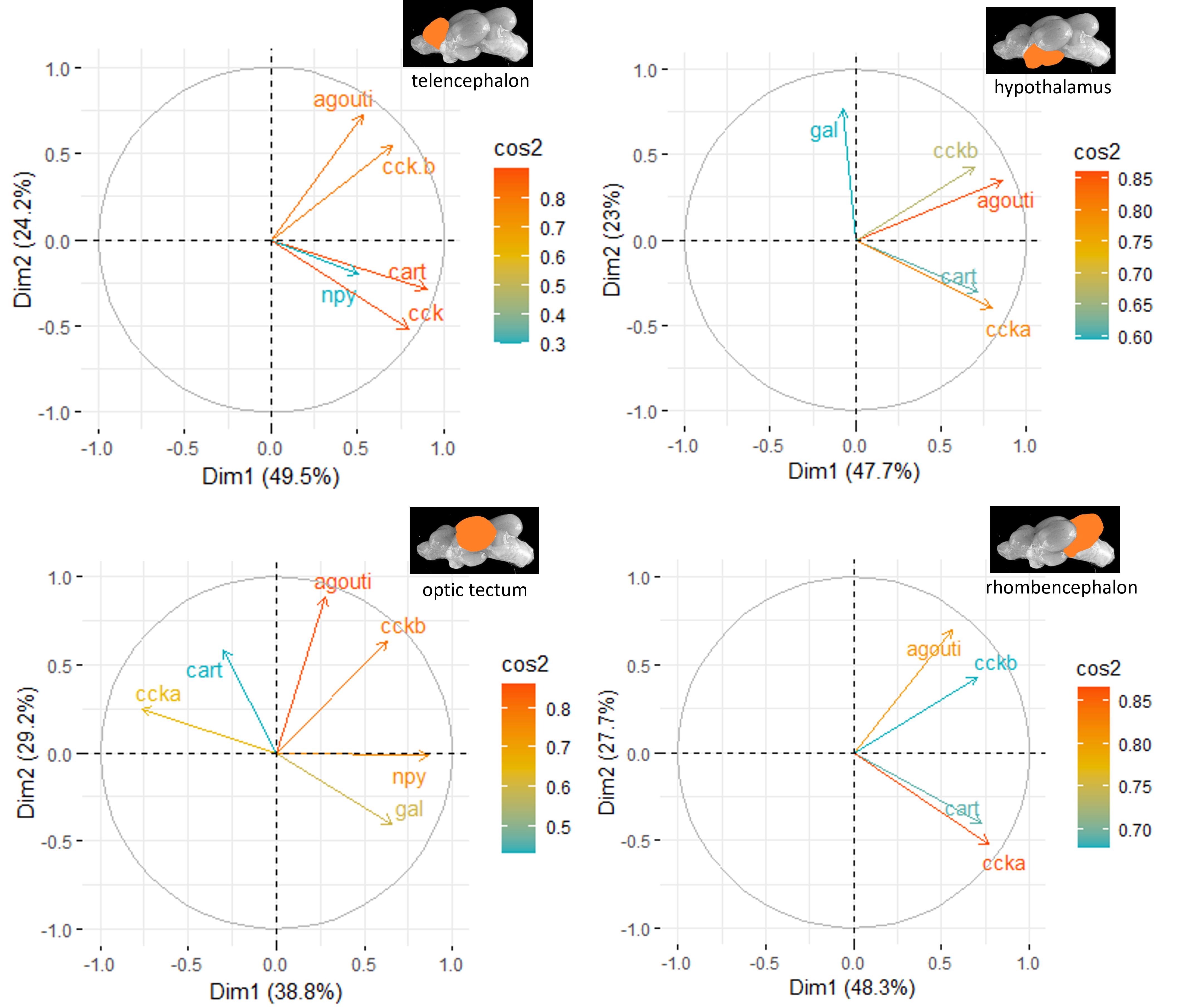


Males


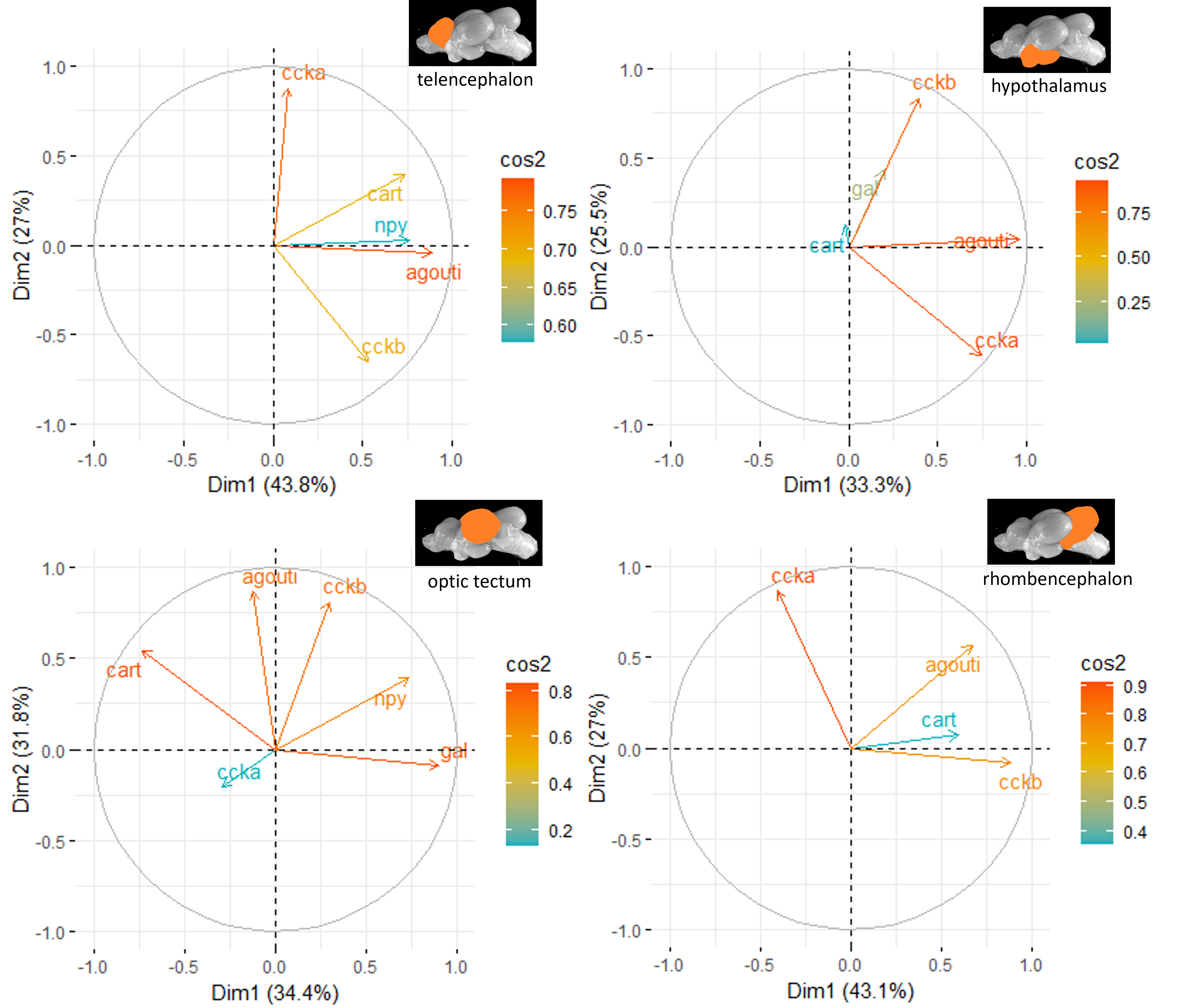


**Figure S4.** Gene expression analysis with Principal Component Analyses (PCA) for the contributing appetite genes in each of the 4 brain parts showing their representation on the factor map as cos2 values, whereby the numbers next to Dim1 and Dim2 indicate the percentage of the variance in the

data sets that is explained by the first two components of the PCA of female (upper 4 figures) and male fish (lower 4 figures); n = 6 per treatment.

Females


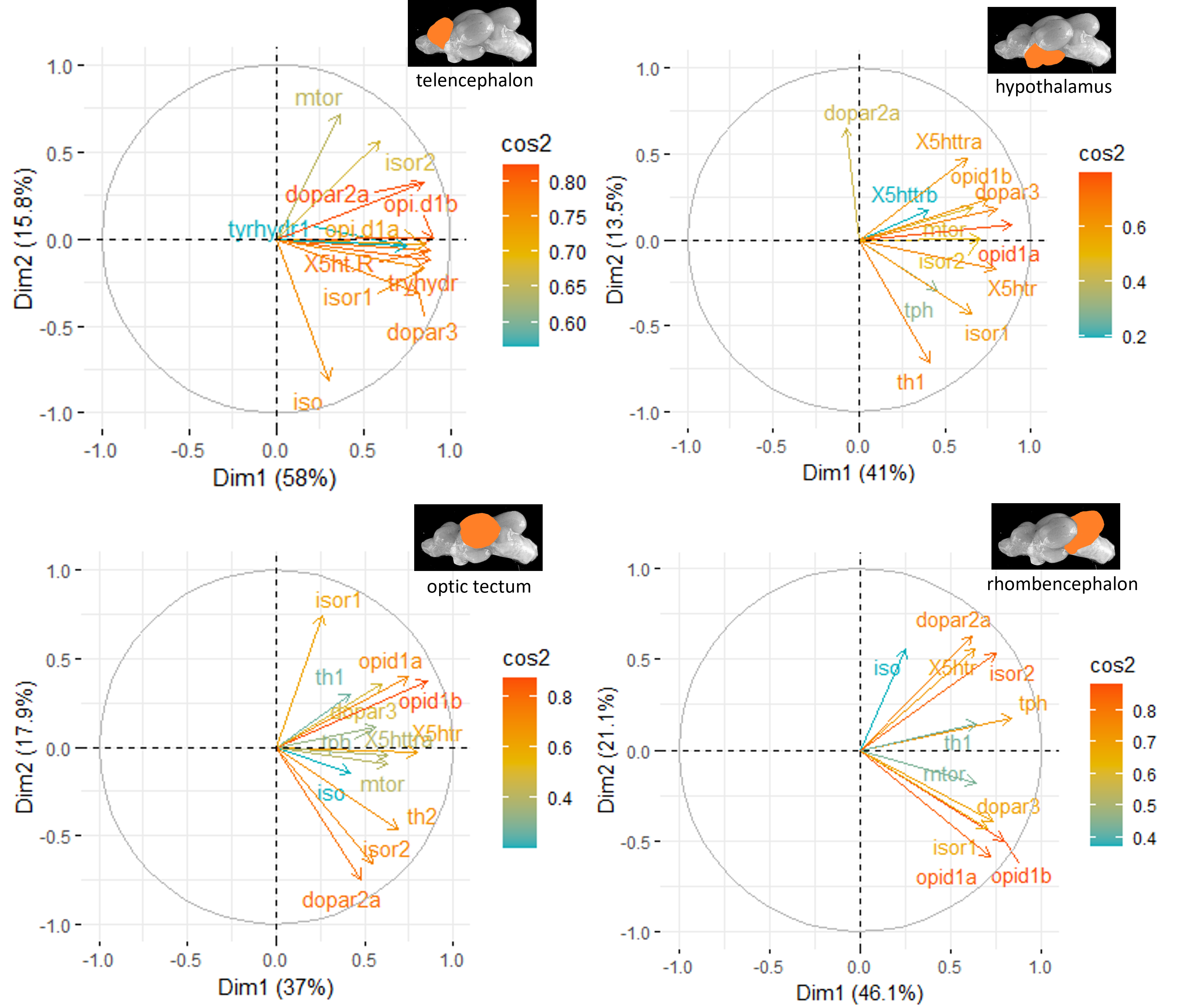


Males


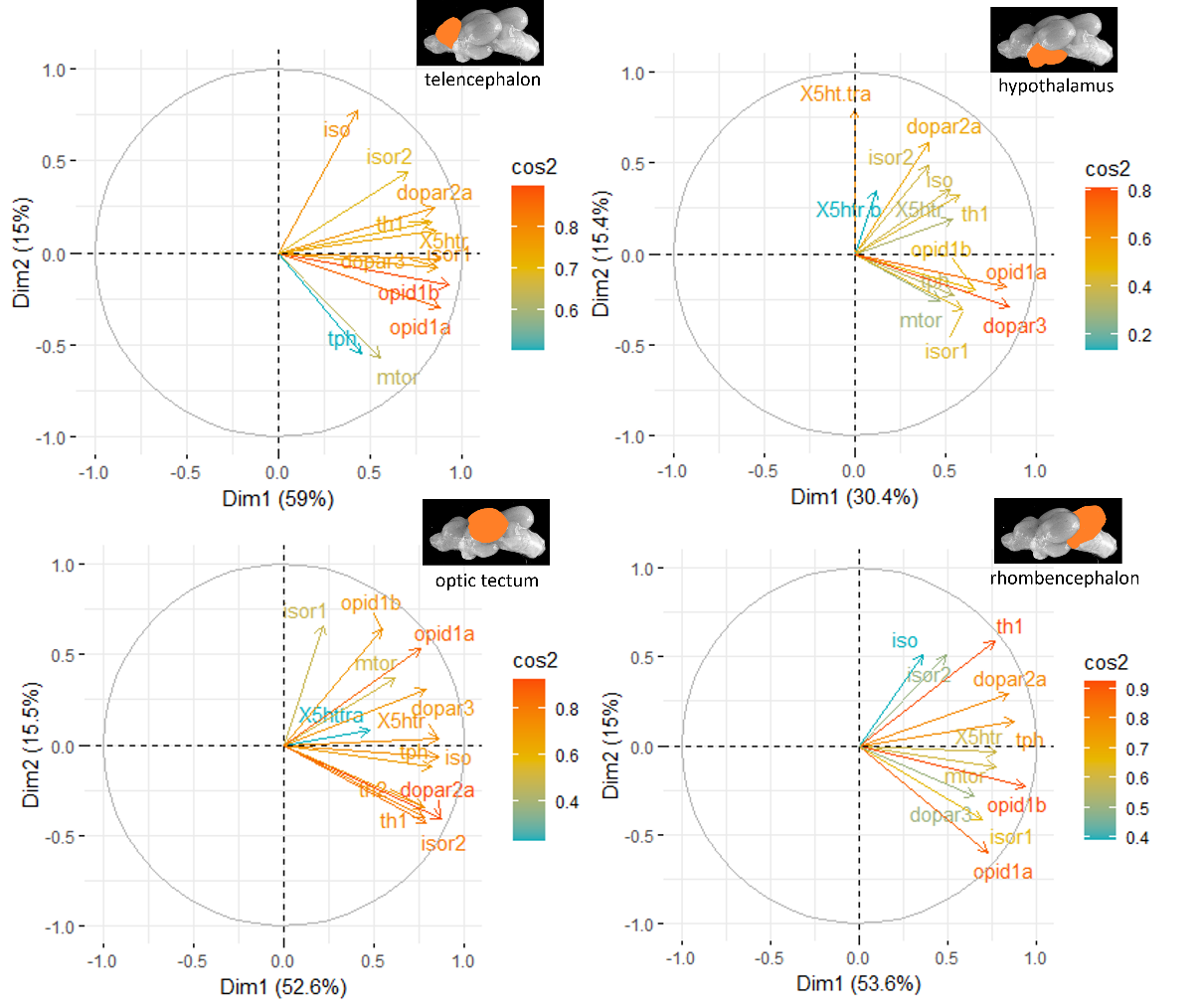


**Figure S5.** Gene expression analysis with Principal Component Analyses (PCA) for the contributing genes belonging to different pathways (e.g., serotonin-, dopamine-, and isotocin-related) in each of the 4 brain parts showing their representation on the factor map as cos2 values, whereby the numbers next to Dim1 and Dim2 indicate the percentage of the variance in the data sets that is explained by the first two components of the PCA of female (upper 4 figures) and male fish (lower 4 figures); n = 6 per treatment.

## Supplementary Tables

**Table S1.** Primer pairs selected for the gene expression studies. The designed primer pairs are assumed to be not able to discriminate between potential transcript variants of the same gene with high sequence similarities.

| **Primer Name** | **Primer Efficiency (%)** | | | **Sense Sequence (5′-3′)** | | **Anti-Sense Sequence (5′-3)** | |
| --- | --- | --- | --- | --- | --- | --- | --- |
| *18S* | | 91 | TGGTGGTGCATGGCCGTTCTT | | TAAGAAGTTGGGACGCCGAC | |  |
| *5ht-r* | | 102 | TCTCACCGTCCAAGCACTTC | | CTGGGAAGAAGGTGGAGCTG | |  |
| *5ht trb* | | 101 | TGTCAAGCAGTTCACCAGACT | | GGGAGAAACTGAAGGAGGCC | |  |
| *agouti* | | 101 | GAGACGCAGAGAAAACTCATTC | | ACAGCTTTGCCCAGATCCTC | |  |
| *bact* | | 110 | GCCGTGACCTGACTGACTAC | | GCCCATCTCCTGCTCAAAGT | |  |
| *cart* | | 103 | GCAAGCGTCATTCCCAGGTG | | GAAGAAAGTGTTGCAGGCGG | |  |
| *cck-a* | | 110 | TGGAGTTCAGTCTAATGTCGG | | TGCGGTATGAGCCTTTGGTT | |  |
| *cck-b* | | 119 | AACTCCTCGATGAACAGCCG | | TGAGTATTCATATTCCTCAGCGC | |  |
| *cfos* | | 111 | ATCGCCAACCTGCTCAAAGA | | AGTGACGATCTCTGGGACTG | |  |
| *ckap* | | 104 | TCGGACACTGAAGACACTGC | | GTTTGACCACTCGCCTCAGA | |  |
| *citrsyn* | | 101 | GAGCCACAGTTCACAGAGCT | | TGAAGGAGAGGTAGGGGTCG | |  |
| *crf1* | | 101 | TTCCACCGCCGTATGAATGT | | CGAGCCGGATGAAGTACTCC | |  |
| *crf-r1* | | 107 | CCAGAGGGCAGTTTCACCAT | | CTGTCCAAGGCTGAGCTCTC | |  |
| *crf-r2* | | 108 | AATTCAGAGGGGTGGCATGG | | CCCAATCCAATTCAGCTACACC | |  |
| *crhbp* | | 120 | GTGTCTGGATATGGTGGCCG | | TTAATGAAATCTCCTCCTCTGC | |  |
| *dopar 2a* | | 101 | GGGACTTCAATGCCACGGAA | | AGTTGGTGGTGGTCTGCAAA | |  |
| *ef* | | 106 | CCACCGGCCATCTGATCTAC | | ACACCCAGGCGTACTTGAAG | |  |
| *eiFa* | | 106 | GGCGTGTTATGGATATGAAGGC | | CCTTCTGTTCGCTCCAGTGT | |  |
| *egr-1* | | 100 | CTTACACAGGCCGTTTCACC | | GAGGAAGAGGGGTGAGTTGC | |  |
| *erk-1* | | 108 | GATCAATGACATCCTGAGAGCT | | CAGAAAGTAGCAGATATGGTC | |  |
| *gal* | | 96 | TCCTCTGATGCTCCTGTGGA | | AAATGTGCTCTGCTCGGTGA | |  |
| *gapdh* | | 106 | CATCCTGCACCACTAACTGC | | GTCTTCTGTGTGGCGGTGTA | |  |
| *gr* | | 103 | TCAGTGGAATGCAGCAGAGG | | GTGTGTGCCAGTCTTTCCAC | |  |
| *iso pre* | | 106 | UGUUUCGGCCCCAGTATCTGC | | GCCTTTCCAGACATCTCACA | |  |
| *iso-r1* | | 106 | GAAACCTGTGCGTCCTTGTG | | GGACTTGAAAAACCGCCACC | |  |
| *iso-r2* | | 98 | TGAATCCCCTCAAGCGGAAC | | TGAAGTAATACATGCGAGACTGG | |  |
| *mr* | | 103 | ATGGAAAGTGCGTCGCTGAGG | | GTTGTGCTGACCTTCTACCGC | |  |
| *neuro d2* | | 94 | TCGAACTGGACCGACAAGTG | | TCCTCTTCTTCTTCCTCGTCG | |  |
| *npy* | | 103 | CCAACAAAACCCGACAACCC | | GGAAGAATTTGAGACTACGCT | |  |
| *opio d1a* | | 104 | CAAGACGCAACTTTCCAGCC | | CATCAGGAACGAAATCAAAACG | |  |
| *opio d1b* | | 117 | TCATCTGCGTTGTGGGACTC | | GGAAGGGCAATGTACTGGTGG | |  |
| *palld* | | 101 | ATCTCCACCTCTCCCACCTC | | TGCGAGCTGTATCCGATGGG | |  |
| *pomc A* | | 103 | GCAAACGCAGACCCATCAAG | | CTCGTTATTTGCCAGCTCGC | |  |
| *pomc B* | | 103 | GTCAAAAACAACGGGAAGTATCG | | TCAGGGGTTTGTGGGATTCG | |  |
| *pyrkin* | | 119 | TGAGATCAGAACTGGGCTTA | | GTTCTTGTAGTCCAACCACAGG | |  |
| *succdh* | | 110 | AGCAGGGGAAGCAGCAGTAC | | GAGAGTCGATCATCCAGCGG | |  |
| *tph* | | 106 | GTCACAGCACAGACCCTCTC | | CTTTTGCACATCTTCGTCTG | |  |
| *tub* | | 114 | GCCTTACAACTCCATCCTGACC | | CGCTCAATGTCAAGGTTCCT | |  |
| *th1* | | 103 | AGCAGCTCCACATCTTCCAC | | TCCTCGATCAAACTCTGCCG | |  |
| *th2* | | 112 | CTGTAAGCAAAACGGAGCAGT | | CTGATCTTGGTATGGTTGAACTGC | |  |
| *uro1* | | 105 | AGCAGGACAACAGCTTGGAG | | CTATTTCCCAACCTCGTCGA | |  |

*18S RNA*: based on FJ915075 for zebrafish (*Danio rerio*) 18S small subunit ribosomal RNA, patrial sequence; *5ht-r:* based on the NM_001129893 for zebrafish 5-hydroxytryptamine receptor 2C, G-protein coupled-like 1;*5ht trb:* based on the sequence for zebrafish (DQ285099) for the serotonin transporter b; *agouti*: based on the sequence for the agouti related neuropeptide (NM_001328012); *bactin*: based on the sequence for beta actin of zebrafish (AF025305); *cart:* based on the cocaine- and amphetamine-regulated transcript 4 for zebrafish (NM_001082932); *cck-a:* based on the predicted sequence for zebrafish cholecystokinin a (XM_001346104); *cck-b*: based on the predicted sequence for zebrafish cholecystokinin b (XM_002665615); *c-fos*: based on the complete cds for zebrafish cfos (DQ003339); *ckap:* based on the sequence for the cytoskeleton associated protein 5 of zebrafish (NM_001037667.3); *citrsyn:* based on the sequence for the citrate synthase of zebrafish (BC045362); *crf1*: based on the sequence XM_009298729.3 for the corticotropin releasing hormone of zebrafish; *crf-r1:* based on the sequence XM_691254.6 for zebrafish; *crf-r2:* based on the sequence for the corticotropin releasing hormone receptor 2 (NM_001113644); *crh-bp:* based on the sequence for the corticotropin releasing hormone-binding protein (BC164122) of zebrafish; *dopar 2a:* based on the sequence NM_183068 available for the dopamine receptor D2a of zebrafish; *ef:* based on the complete sequence ZEFEF1AL for the elongation factor 1 alpha of zebrafish; *eiFa:* based on the complete sequence for the eIF2 alpha subunit of zebrafish; *erk1:* based on the complete cds for zERK1 (AB030902); *gal:* based on the sequence for the galanin/GMAP prepropeptide for zebrafish (galn – 100006361); *gapdh:* based on the partial cds for the glyceraldehyde-3-phosphate dehydrogenase of zebrafish (AY818347); *gr:* based on the sequence for the nuclear receptor subfamily 3 (nr3c1, glucocorticoid receptor) of zebrafish (NM_001020711); *iso pre:* based on the sequence for isotocin of zebrafish (AY069956); *iso-r1:* based on the sequence for the isotocin receptor-like 1 mRNA for zebrafish (FJ556869); *iso-r2:* based on the sequence for the isotocin receptor-like 2 mRNA for zebrafish (FJ556870); *mr:* based on the sequence for the nuclear receptor subfamily 3, group C, member 2 (nr3c2, mineralcorticoid receptor) of zebrafish (NM_001100403); *neurod*: based on the complete cds for neuroD of zebrafish (AF017302); *npy:* based on the cds for the neuropeptide y of zebrafish (NM_131074); *opio d1a:* based on the sequence NM_131258 for the zebrafish opioid receptor delta 1a; *opio d1b:* based on the sequence NM_212755 for the zebrafish opioid receptor delta 1b; *palld:* based on the predicted sequence for palladin, the cytoskeletal associated protein, transcript variant X1 (XM_021468620; *pomc A*: based on the sequence for the proopiomelanocortin a mRNA of zebrafish (BC133874); *pomc B*: based on the predicted *Danio rerio* proopiomelanocortin b transcript variant X1 (XM_005170169.4; *pyrkin:* based on the *Danio rerio* pyruvate kinase mRNA (BC165710); *succdh*: based on the succinate dehydrogenase complex subunit D integral membrane protein a mRNA of zebrafish (BC079507); *tph:* based on the tryptophane hydroxylase 2 of zebrafish (NM_001310068); *th1:* based on the *Danio rerio* tyrosine hydroxylase mRNA of zebrafish (NM_131149); *th2:* based on the tyrosine hydroxylase mRNA of *Danio rerio* (NM_001001829); *tub:* based on the sequence for the zebrafish tubulin alpha 1a (NM_001190982); *uro1:* based on the sequence for the *Danio rerio* urotensin 1 mRNA (BC124468).

**Table S2.** Comprehensive gene stability as assessed by RefFinder (http://blooge.cn/RefFinder/) based on the algorithms of the Delta CT method, the Best Keeper method, normFinder, and GeNorm.

| **Brain Part** |  | **Geometric mean of the ranking values from each individual method** |
| --- | --- | --- |
| *telencephalon* |  | gapdh: 1.32 / ckap5: 1.41 |
| *hypothalamus* |  | ckap5: 1.00 / succdh: 2.11 |
| *optic tectum* |  | succdh: 1.41 / ckap5: 1.73 |
| *rhombenecephalon* |  | ckap5: 1.19 |

**Table S3**. ANOVA Tables for the morphological description of the fish assuming treatment group (group), gender, and an interaction of group and gender, TL = total body length, SL = standard body length, condition = Fulton`s condition factor, bmi = body mass index, n = 6 for each group.

**Response: TL**

Sum Sq Df F value Pr(>F)

(Intercept) 95.202 1 1780.8574 < 2e-16 ***

group 0.181 3 1.1302 0.34842

gender 0.163 1 3.0553 0.08815

group:gender 0.082 3 0.5131 0.67557

Residuals 2.138 40

**Response: SL**

Sum Sq Df F value Pr(>F)

(Intercept) 62.727 1 1701.0621 <2e-16 ***

group 0.088 3 0.7947 0.5041

gender 0.067 1 1.8305 0.1837

group:gender 0.052 3 0.4727 0.7030

Residuals 1.475 40

**Response: weight**

Sum Sq Df F value Pr(>F)

(Intercept) 2947004 1 181.7439 < 2.2e-16 ***

group 44458 3 0.9139 .442864

gender 160314 1 9.8867 0.003135 **

group:gender 5476 3 0.1126 0.952246

Residuals 648606 40

**Response: condition**

Df Sum Sq Mean Sq F value Pr(>F)

group 3 0.0848 0.0283 2.536 0.0703 .

gender 1 0.6008 0.6008 53.931 6.26e-09 ***

group:gender 3 0.0332 0.0111 0.995 0.4052

Residuals 40 0.4456 0.0111

**Response: bmi**

Df Sum Sq Mean Sq F value Pr(>F)

group 3 0.000193 0.000064 1.658 0.191

gender 1 0.003554 0.003554 91.576 6.76e-12 ***

group:gender 3 0.000042 0.000014 0.358 0.783

Residuals 40 0.001552 0.000039

**Table S4**. ANOVA Tables for the morphological description of the fish assuming treatment group (group), gender, and an interaction of group and gender, n = 6 for each group.

| Model: cortisol ~ group * gender | | |  |  |  |  |
| --- | --- | --- | --- | --- | --- | --- |
|  |  |  |  |  |  |  |
|  | Df | Sum Sq | Mean Sq | F value | Pr(>F) |  |
| group | 3 | 22.75 | 7.585 | 4.21 | **0.012** | * |
| gender | 1 | 16 | 15.998 | 8.881 | **0.005** | ** |
| group:gender | 3 | 0.76 | 0.252 | 0.14 | 0.936 |  |
| Residuals | 36 | 64.85 | 1.801 |  |  |  |
|  |  |  |  |  |  |  |

| Model: cortisone ~ group * gender | | | |  |  |  |
| --- | --- | --- | --- | --- | --- | --- |
|  |  |  |  |  |  |  |
|  | Df | Sum Sq | Mean Sq | F value | Pr(>F) |  |
| group | 3 | 21.84 | 7.281 | 5.608 | **0.0029** | ** |
| gender | 1 | 23.08 | 23.08 | 17.775 | **0.0002** | *** |
| group:gender | 3 | 1.62 | 0.541 | 0.417 | 0.7422 |  |
| Residuals | 36 | 46.74 | 1.298 |  |  |  |
